# Mitigating Sociodemographic Bias in Opioid Use Disorder Prediction: Fairness-Aware Machine Learning Framework

**DOI:** 10.2196/55820

**Published:** 2024-08-20

**Authors:** Mohammad Yaseliani, Md Noor-E-Alam, Md Mahmudul Hasan

**Affiliations:** 1 Department of Pharmaceutical Outcomes and Policy, College of Pharmacy, University of Florida Gainesville, FL United States; 2 Department of Mechanical and Industrial Engineering, Northeastern University Boston, MA United States; 3 The Institute for Experiential AI, Northeastern University Boston, MA United States; 4 Department of Information Systems and Operations Management, Warrington College of Business, University of Florida Gainesville, FL United States

**Keywords:** opioid use disorder, fairness and bias, bias mitigation, machine learning, majority voting

## Abstract

**Background:**

Opioid use disorder (OUD) is a critical public health crisis in the United States, affecting >5.5 million Americans in 2021. Machine learning has been used to predict patient risk of incident OUD. However, little is known about the fairness and bias of these predictive models.

**Objective:**

The aims of this study are two-fold: (1) to develop a machine learning bias mitigation algorithm for sociodemographic features and (2) to develop a fairness-aware weighted majority voting (WMV) classifier for OUD prediction.

**Methods:**

We used the 2020 National Survey on Drug and Health data to develop a neural network (NN) model using stochastic gradient descent (SGD; NN-SGD) and an NN model using Adam (NN-Adam) optimizers and evaluated sociodemographic bias by comparing the area under the curve values. A bias mitigation algorithm, based on equality of odds, was implemented to minimize disparities in specificity and recall. Finally, a WMV classifier was developed for fairness-aware prediction of OUD. To further analyze bias detection and mitigation, we did a 1-N matching of OUD to non-OUD cases, controlling for socioeconomic variables, and evaluated the performance of the proposed bias mitigation algorithm and WMV classifier.

**Results:**

Our bias mitigation algorithm substantially reduced bias with NN-SGD, by 21.66% for sex, 1.48% for race, and 21.04% for income, and with NN-Adam by 16.96% for sex, 8.87% for marital status, 8.45% for working condition, and 41.62% for race. The fairness-aware WMV classifier achieved a recall of 85.37% and 92.68% and an accuracy of 58.85% and 90.21% using NN-SGD and NN-Adam, respectively. The results after matching also indicated remarkable bias reduction with NN-SGD and NN-Adam, respectively, as follows: sex (0.14% vs 0.97%), marital status (12.95% vs 10.33%), working condition (14.79% vs 15.33%), race (60.13% vs 41.71%), and income (0.35% vs 2.21%). Moreover, the fairness-aware WMV classifier achieved high performance with a recall of 100% and 85.37% and an accuracy of 73.20% and 89.38% using NN-SGD and NN-Adam, respectively.

**Conclusions:**

The application of the proposed bias mitigation algorithm shows promise in reducing sociodemographic bias, with the WMV classifier confirming bias reduction and high performance in OUD prediction.

## Introduction

### Background

Opioid use disorder (OUD) and opioid overdose (OD) continue to remain a major public health crisis in the United States. OUD significantly contributes to overdoses, and >81,000 individuals lost their lives because of OD from April 2021 to April 2022 [1-3]. Meanwhile, the COVID-19 pandemic has worsened the ongoing OUD and OD epidemic [4]. In addition, the economic burden of OUD in the United States is overwhelming, with an estimated annual cost exceeding US $786 billion in 2018 [1]. Therefore, it is critical to design interventions to facilitate an informed prescribing practice and monitoring of opioids to reduce the prevalence of OUD and subsequent drug overdose deaths.

Previous studies have used conventional regression-based methods to identify the significant predictors of OUD and OD [5-16]. More recently, machine learning (ML) methods have shown great potential in developing reliable predictive tools for identifying individuals at higher risk of OUD and OD [17]. ML methods can handle the complex nonlinear relationship among predictors and outcomes and perform well on imbalanced data [18]. Using different features as inputs, ML can predict the risk of developing OUD and OD with higher predictive accuracy [19,20]. Previous studies have developed random forests [16,21-24], decision trees [22,24], gradient boosting [16,23,25,26], neural networks (NNs) [5,16,27,28], and long short-term memory networks [24,28,29] to predict OUD and OD with impressive predictive performance.

Prior studies also reported that the risk of OUD or OD varies based on several individual-level protected sociodemographic features [30-32], potentially causing user-related bias. For instance, economically disadvantaged areas present higher levels of opioid use compared to other areas. Studies have highlighted significant sex differences in OUD in the United States, with women experiencing higher rates of prescription opioid use and faster progression to dependency compared to men [33,34]. In addition, opioid prescribing is 2 times more likely for White individuals than Black individuals in the United States [35,36], attributed to physicians’ practice biases [37]. Therefore, the real-world data describing OUD patterns often include biases caused by users. These biases, alongside potential sampling or algorithmic biases, could cause unfair and biased outcomes, leading to suboptimal model performance and inequalities in patient care [38].

### Objectives

In this study, we aim to address the limitations of prior studies, including the lack of attention to (1) detecting and analyzing the fairness and bias in the ML or deep learning (DL) models for predicting OUD and (2) proposing methods to mitigate bias for different protected attributes. Using the data provided by the National Survey on Drug Use and Health (NSDUH) for 2020, we developed an NN model to detect the bias for different sociodemographic features [39,40]. We then propose an algorithm based on equality of odds (EO) to mitigate the bias while ensuring reasonable predictive performance (ie, accuracy and recall). Finally, we create a fairness-aware weighted majority voting (WMV) classifier that considers the predicted classes using the optimal thresholds for different sociodemographic features and outputs the most frequent class. To show the effectiveness of the proposed methods, we also evaluate their performance by developing several ML algorithms, including logistic regression (LR), linear support vector machine (SVM), and SVM–radial basis function (SVM-RBF).

## Methods

### Data and Sample

We used the 2020 NSDUH survey data that was conducted using an independent multistage area probability design [41]. The study sample included a community-based noninstitutionalized population aged ≥12 years, with information on clinical characteristics, sociodemographic factors, and substance use. The final data included 32,893 individuals with 2892 variables for public use.

### Features and Outcome Variable

We selected the features based on the prior research identifying predictors of OUD [42-52]. The sociodemographic features included sex, marital status, working condition (whether someone works ≥35 h/wk), race (Black, White, and other racial groups), and income (<US $20,000 per year and other groups). We also included a history of using different types of prescription opioids [42] (eg, oxycodone, oxymorphone, hydrocodone, hydromorphone, fentanyl, morphine, codeine, methadone, tramadol, and buprenorphine), use of heroin, history of receiving alcohol or drug treatment, diabetes [43], chronic bronchitis [44], cirrhosis of the liver [45], hepatitis B or C [46,47], kidney disease [48], asthma [49], AIDS [50], cancer [51], depression [52], and BMI [53,54]. A total number of 26 features were included in the proposed ML and DL models. After one-hot encoding, there were a total of 44 features, including BMI. These features are presented in Multimedia Appendix 1.

As an outcome variable, we used whether an individual has developed OUD, which is defined as the dependence, misuse, and abuse of opioids [55]. To train the classifiers, we used stratified 80:20 train-test splitting. Of the 26,314 individuals, the training set included 26,148 (99.37%) and 166 (0.63%) individuals belonging to non-OUD and OUD classes, respectively. Moreover, of the 6579 individuals, there were 6538 (99.38%) non-OUD and 41 (0.62%) OUD individuals in the test set.

### Classifiers

To perform this prediction task, we primarily developed and evaluated 3 well-known ML or DL models: LR [56], SVM [57], and NN [58]. We first designed an NN model consisting of 4 layers, in which the first layer takes 44 features as inputs, the 2 hidden layers include 1000 neurons, and the last layer consists of 1 neuron for making predictions. We implemented this NN model using the TensorFlow library in Python created by Abadi et al [59] using minibatch stochastic gradient descent (SGD) and the Adam optimizer, using the default learning rate of 0.001. Minibatch SGD was chosen to optimize the convergence speed and computational efficiency, as it balances the stability of full-batch gradient descent and the high variance of SGD, enabling more robust learning [60]. The Adam optimizer was used for its adaptive learning rate properties, which adjust the learning rate for each parameter individually. This optimizer combines the benefits of AdaGrad and RMSProp, making it effective for handling sparse gradients and nonstationary objectives [61]. A batch size of 64 was used to leverage parallelism on modern hardware, thus reducing computational time and improving scalability. The NNs were trained for 20 epochs using the binary cross-entropy loss function with balanced class weighting to address any class imbalance in the data set, ensuring that the model is not biased toward the majority class and performs well on minority class instances.

As noted before, we also trained ML classifiers to test the performance of the proposed methods. To train the LR classifier, we fitted intercept and used the balanced class weighting to prevent overfitting or underfitting. To train the SVM classifiers, we used a *C* value of 1 with balanced class weighting. The default *γ* value for the RBF kernel in the SVM-RBF classifier was calculated according to equation 1:



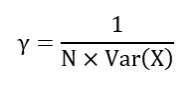



**(1)**

Notably, we used recall, specificity, and accuracy to assess the performance of all these models.

### Bias Detection Based on the Area Under the Curve Values

We largely followed the study by Fletcher et al [39] to detect both the algorithmic and sampling bias for each of the sociodemographic features (ie, sex, marital status, working condition, race, and income) based on the area under the curve (AUC) value. We computed the AUC values for each category of a sociodemographic feature, where any difference in the AUC values identified the presence of algorithmic bias. This type of bias is the result of internal model specifications and features [39].

Sampling bias detection and mitigation are critical in ensuring the fairness and effectiveness of ML models, especially in the context of global health. As discussed in the study by Fletcher et al [39], sampling bias arises when the data used to train an ML model does not adequately represent the actual proportions found in the real world. This can lead to models that perform poorly on minority groups and introduce unfairness into the decision-making process. The methodology for detecting sampling bias involves creating homogenous test groups for each demographic category and comparing the model’s performance. By examining the AUC and the variability of model performance across these groups, the biases caused by sampling can be identified. For instance, if a model trained predominantly on data from one demographic group consistently underperforms when applied to another group, this indicates the presence of sampling bias. Such disparities highlight the model’s inability to generalize well across diverse populations, which is a fundamental flaw in its design. In our study, we systematically tested for sampling bias by examining the model’s performance across different demographic groups.

To detect the sampling bias, we first created training sets consisting of different compositions of individuals belonging to each sociodemographic group. We then computed and plotted the models’ AUC value for each category based on a fixed-size test set. A significant fluctuation in the models’ AUC values with respect to the change in the structure of the training sets indicated the presence of the sampling bias for a sociodemographic feature [39].

In this study, we analyzed 5 sociodemographic features, including sex, marital status, working condition, race, and income, to detect the algorithmic bias. The categories included male individuals and female individuals, those who have never been married and other groups related to marital status, those who work ≥35 hours per week and other groups associated with working condition, Black and White race, and those who have an income of <US $20,000 per year and others. To detect the sampling bias, we created a test set of fixed size for each sociodemographic feature: sex: 5984 (2992 male individuals and 2992 female individuals), marital status: 5648 (2824 from *never been married* and 2824 from other groups), working condition: 5296 (2648 from *working ≥35 hours* and 2648 from other groups), race: 1280 (640 Black race and 640 from White race), and income: 2000 (1000 from *income <US $20,000* and 1000 from other groups). These test sets were created based on the main test set used during model development (6579/32,893, 20% of the whole data).

To further enhance the validity of the proposed predictive model, we implemented a detailed 1-N matching process, in which we controlled for a variety of socioeconomic variables, including BMI, sex, marital status, working condition, race, and income, to achieve a balanced comparison between OUD and non-OUD cases. To ensure robust matching, we used a 1-N matching strategy with n=158, meaning each OUD case was matched with 158 non-OUD cases. This number was calculated based on the proportion of OUD-negative to OUD-positive cases in the 2020 NSDUH data used in this study. Following 1-N matching, the training set included 26,164 non-OUD and 166 OUD individuals. Similarly, 6542 individuals belonged to the non-OUD class in the test set and 41 were in the OUD class. Accordingly, we implemented the same sampling and algorithmic bias detection approaches on the matched data to comprehensively analyze the existence of bias in the predictive models. This extensive matching allowed us to control potential confounders and provide a more accurate data set for our analysis.

### The Proposed Method for Bias Mitigation

To propose a method for bias mitigation, we considered a fairness definition called EO [62], which is measured based on the difference between the specificity and recall or sensitivity values. This approach is grounded in the study by Hardt et al [62], in which their framework introduces a robust criterion for measuring and removing discrimination based on protected attributes, emphasizing that fairness can be optimized post hoc through threshold adjustments. They proposed that any learned predictor can be adjusted to achieve nondiscrimination by modifying its thresholds, ensuring that the predictor’s true positive rates (recall) and false positive rates (1–specificity) are independent of the protected attribute, thereby satisfying the EO criterion. This framework suggests that EO can be achieved without altering the underlying complex training pipeline of the predictor. Instead, a simple postprocessing step is sufficient, which is both practical and efficient. This method is robust to changes in class distribution and ensures that the model remains fair by balancing the sensitivity and specificity across different groups. By focusing on minimizing the difference between recall and specificity, we ensure that our model does not favor one group over another, thereby maintaining fairness and robustness in our predictions. The postprocessing adjustment of thresholds allows us to achieve these fairness criteria without compromising the utility of the model, providing a balanced approach to bias mitigation. Equation 2 demonstrates the formula of EO,



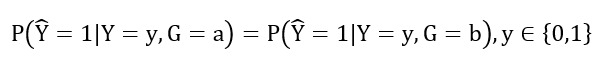



**(2)**

where *G* denotes the group being analyzed and *y* represents the output class. This is equivalent to balancing the recall and specificity values of both groups and considering them equal.

To achieve the EO, we change the classification threshold [62] so that the difference between the recall and specificity is minimized. To address the decreased performance measures (ie, recall and accuracy) because of the threshold moving, we define minimum values for recall and accuracy to ensure that they are above a certain value while changing the classification threshold (70% for recall and 50% for accuracy). We tested the threshold values in the range (0, 100) and identified the optimal one where the recall and accuracy constraints are satisfied and the difference between specificity and recall is minimum. Algorithm 1 (Textbox 1) presents the details of the proposed bias mitigation method. The input is a trained ML or DL model for OUD prediction, and the output is an optimal threshold value based on which the classifications are performed.

**Algorithm 1.**

Input: A trained machine learning (or deep learning)–based model for the prediction of opioid use disorder.

Output: An optimal threshold value.

Begin

1 Z_Values = []

2 th_Values = []

3 th ← 0

4 i ← 0

5 While th ≤ 100 do

6 Calculate the overall recall and accuracy of the model.

7 If recall ≥0.7 and accuracy ≥ 0.5 then

8 Calculate x_1_ and x_2_ (specificity values), and y_1_ and y_2_ (recall values)

9 Calculate 
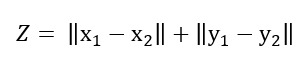

10 Append (Z_Values, Z)

11 Append (th_Values, th)

12. th ← th + 0.1

13 For *i* = 1 to 1001 do

14 if Z_Values[i] = Min(Z_Values) then

15 Best_Threshold th_Values[i]

End

### The Proposed WMV Classifier

Algorithm 1 (Textbox 1) will output different classification thresholds for each sociodemographic feature. Therefore, we may achieve different outputs or classes for a given individual using those thresholds. However, in most cases, we need to have a predictive model that takes into account the bias-related issues for multiple sociodemographic features and predicts OUD for a given individual such that the bias is mitigated to the greatest extent. In this regard, we propose a WMV classifier, which yields a class based on the classifications by each threshold according to equation 3,

*P = w_1_ × pc_1_ + w_2_ × pc_2_ + ... + w_n_ × pc_n_* **(3)**

where *P* is representative of the final probability, *pc_i_* shows the class predicted (ie, 0 and 1) using the threshold for a sociodemographic feature *I*, and *w_i_* shows the weight assigned to feature *i*. To assign a weight to each feature, we calculate the proportion of differences between recall and specificity to gain a value in the range [0,1] and acquire the final probability using equation 3.

### Ethical Considerations

This study did not require approval from an institutional ethics board or review committee as it did not involve any human participants or identifiable personal data. The study used publicly available, anonymized data sets that are confidential and only used for statistical purposes per federal law.

## Results

### Overview

We considered the NN models trained with SGD and Adam optimizers (NN) model using SGD [NN-SGD] and NN model using Adam [NN-Adam]) as the main classifiers and reported results that we obtained for bias detection and mitigation for predicting OUD. We also described the results for 3 ML classifiers (ie, LR, linear SVM, and SVM-RBF) in Multimedia Appendix 2.

### Individual Characteristics

The individuals in the training (26,314/32,893, 80%) and test (6579/32,893, 20%) samples had similar sociodemographic and clinical features (Multimedia Appendix 1). The mean BMI of individuals was 25.58 (95% CI 25.49-25.68; SD 6.68), 54% (17,763/32,893) of individuals were female, and 46% (15,130/32,893) had developed OUD. While the least used opioid was oxymorphone (95/32,893, 0.29%), the most commonly used opioids were hydrocodone (3495/32,893, 10.63%), followed by oxycodone (2147/32,893, 6.53%) and codeine (2014/32,893, 6.12%). In addition, approximately 4.87% (1602/32,893) of individuals had the experience of receiving drug treatments. In total, 28.82% (9482/32,893) of individuals had a history of depression, followed by asthma (3934/32,893, approximately 11.96%) and diabetes (1848/32,893, 5.62%). In addition, >36.93% (12,146/32,893) of individuals were married, and approximately 40.77% (13,409/32,893) of individuals worked for ≥35 hours per week. In addition, approximately 9.19% (3025/32,893) of individuals were Black, 64.94% (21,362/32,893) were White, and the rest belonged to other races (8506/32,893, 25.86%). Furthermore, while approximately 14.95% (4917/32,893) of individuals had an income of <US $20,000 per year, almost 85.05% (27,976/32,893) earned >US $20,000 yearly. Table 1 summarizes the sociodemographic features used in the study.

**Table 1:** The details of sociodemographic variables in the study (N=32,893)^a^.

Sociodemographic variables			Total		Non-OUD^b^ (n=32,686, 99.37%)		OUD (n=207, 0.63%)
**Sex, n (%)**							
	Male	15,130 (46)		15,024 (45.96)		106 (51.21)	
	Female	17,763 (54)		17,662 (54.04)		101 (48.79)	
**Marital status, n (%)**							
	Married	12,146 (36.93)		12,101 (37.02)		45 (21.74)	
	Widowed	661 (2.01)		659 (2.02)		2 (1)	
	Divorced or separated	2613 (7.94)		2569 (7.86)		44 (21.26)	
	Never been married	14,452 (43.94)		14,346 (43.89)		106 (51.21)	
**Working condition, n (%)**							
	>35 hours per week	13,409 (40.77)		13,356 (40.86)		53 (25.6)	
	<35 hours per week	4644 (14.12)		4619 (14.13)		25 (12.08)	
**Race, n (%)**							
	White	21,362 (64.94)		21,213 (64.9)		149 (71.98)	
	Black	3025 (9.2)		3008 (9.2)		17 (8.21)	
	Other racial groups	8506 (25.86)		8465 (25.9)		41 (19.81)	
**Income (US $), n (%)**							
	<$20,000	4917 (14.95)		4851 (14.84)		66 (31.88)	
	Other income groups	27,976 (85.05)		27,835 (85.16)		141 (68.12)	
BMI (kg/m^2^), mean (SD)			25.59 (8.82)		25.59 (8.82)		25.16 (9.83)

^a^For marital status and working conditions, we excluded those not answering the questionnaire or those who legitimately skipped the question, as described in the National Survey on Drug Use and Health data dictionary.

^b^OUD: opioid use disorder.

### Performance of the Classifiers

**Figure 1:**
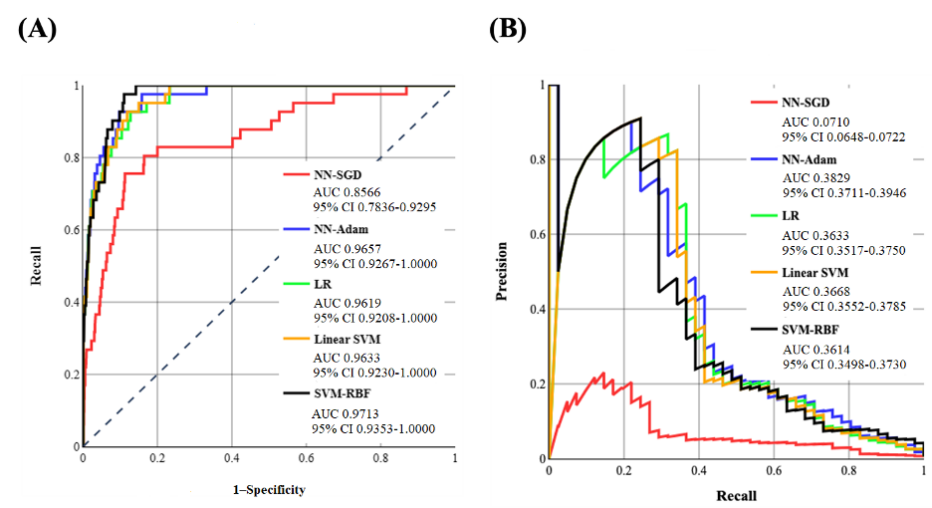
The receiver operating characteristic and precision-recall curves of the classifiers. (A) The receiver operating characteristics curve of the classifiers, (B) precision-recall curve of the classifiers. AUC: area under the curve; LR: logistic regression; NN: neural network; RBF: radial basis function; SGD: stochastic gradient descent; SVM: support vector machine.

Figure 1 shows the receiver operating characteristic (ROC) and precision-recall (PR) curves of the trained ML or DL classifiers for OUD prediction. As shown in Figure 1, while the SVM-RBF classifier has the highest ROC/AUC (AUC 97.13%, 95% CI 93.53%-100%), NN-Adam performs best in terms of PR/AUC (AUC 38.29%, 95% CI 37.11%-39.46%). Moreover, the NN-Adam outperforms the NN-SGD (ROC/AUC 85.66%, 95% CI 78.36%-92.95%; ROC/PR 7.10%, 95% CI 6.48%-7.72%) and NN-Adam (ROC/AUC 96.57%, 95% CI 92.67%-100.00%; ROC/PR 38.29%, 95% CI 37.11%-39.46%).

**Figure 2:**
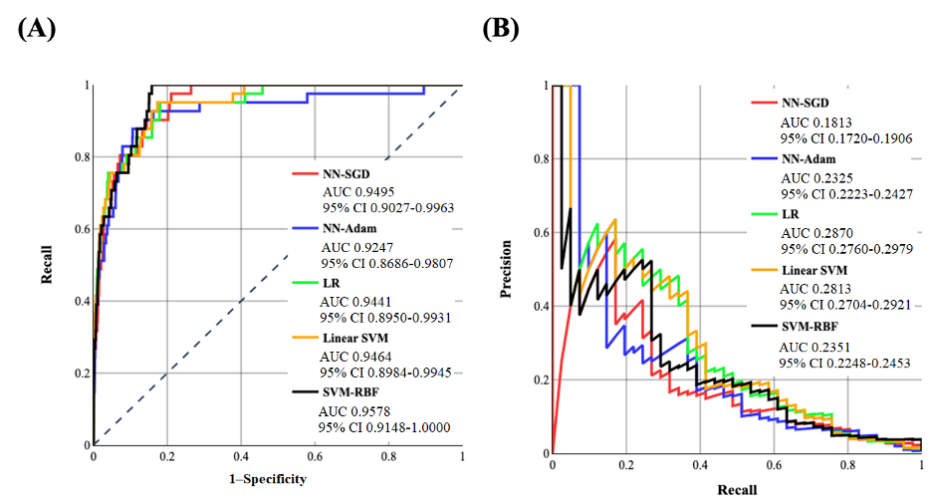
The receiver operating characteristic and precision-recall curves of the classifiers after matching. (A) The receiver operating characteristics curve of the classifiers, (B) precision-recall curve of the classifiers. AUC: area under the curve; LR: logistic regression; NN: neural network; RBF: radial basis function; SGD: stochastic gradient descent; SVM: support vector machine.

Figure 2 shows the ROC and PR curves of the ML or DL classifiers for OUD prediction after matching. As shown in Figure 2, the SVM-RBF classifier has the highest ROC/AUC (AUC 97.13%, 95% CI 93.53%-100%), while LR has the highest PR/AUC (AUC 28.70%, 95% CI 27.60%-29.79%). Moreover, while NN-SGD outperforms NN-Adam in terms of ROC/AUC, NN-Adam has a higher PR/AUC (NN-SGD: ROC/AUC 94.95%, 95% CI 90.27%-99.63%; ROC/PR 18.13%, 95% CI: 17.20%-19.06%; NN-Adam: ROC/AUC 92.47%, 95% CI 86.86%-98.07%; ROC/PR 23.25%, 95% CI 22.23%-24.27%).

Figure S1 in Multimedia Appendix 2 reports the confusion matrices of NN models. While the NN-SGD correctly classifies only 22% (9/41) of the individuals who have developed OUD, the NN-Adam correctly classifies 71% (29/41) of individuals. Moreover, the NN-SGD misclassifies 0.54% (35/6538) of the individuals who have not developed OUD, whereas the NN-Adam misclassifies 2.68% (175/6538) of individuals. Overall, the NN-SGD and NN-Adam achieve a recall of 21.95% and 70.73%, a specificity of 99.46% and 97.32%, and an accuracy of 98.98% and 97.16%, respectively.

The confusion matrices of the ML classifiers, including LR, linear SVM, and SVM-RBF, are presented in Figure S2 in Multimedia Appendix 2. All these classifiers have an accuracy/specificity of >92% and an AUC of >96%. Moreover, the linear SVM classifier has the highest recall of 82.93%, followed by LR and SVM-RBF with 80.49% and 26.83%, respectively.

### 1-N Matching

The standardized mean difference and variance ratio before and after matching for different sociodemographic groups are presented in Table 2.

**Table 2:** The details of 1-N matching (N=158).

Status				SMD^a^		Variance ratio
**Race**						
	**White**					
		Before matching	0.1526		0.8896	
		After matching	0.0188		0.9869	
	**Black**					
		Before matching	–0.0351		0.9065	
		After matching	–0.0385		0.8979	
	**Other**					
		Before matching	–0.1453		0.8317	
		After matching	0.0060		1.0141	
**Income**						
	**<US $20,000 per year**					
		Before matching	0.4106		1.7267	
		After matching	–0.0178		0.9913	
**Sex**						
	**Male**					
		Before matching	0.1049		1.0108	
		After matching	0.0069		1.0045	
**Marital status**						
	**Married**					
		Before matching	–0.3400		0.7332	
		After matching	0.0094		1.0180	
	**Widowed**					
		Before matching	–0.0866		0.4867	
		After matching	-0.0019		0.9863	
	**Divorced or separated**					
		Before matching	0.3862		2.3224	
		After matching	0.0324		1.0534	
	**Never married**					
		Before matching	0.1467		1.0195	
		After matching	–0.0077		1.0053	
**Working condition**						
	**Working ≥35 hours per week**					
		Before matching	–0.3279		0.7921	
		After matching	0.0391		1.0520	
	**Other groups of working condition**					
		Before matching	–0.0608		0.8793	
		After matching	0.0069		1.0212	
**BMI**						
	Before matching			–0.0464		1.2435
	After matching			0.0169		1.0244

^a^SMD: standardized mean difference.

### Bias Detection (Algorithmic Bias)

**Figure 3:**
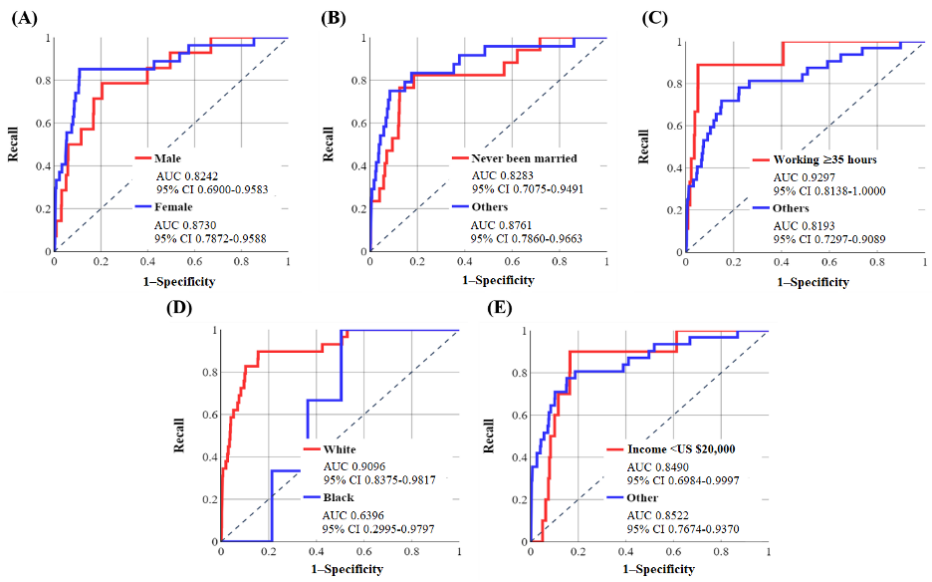
The receiver operating characteristic (ROC) curves for various groups related to sociodemographic features using the neural network model using stochastic gradient descent (with area under the curve [AUC] and 95% CI values). Values were calculated based on the test sample (6579 individuals: 41 developed opioid use disorder [OUD] and 6538 did not develop OUD). (A) ROC curve for sex, (B) ROC curve for marital status, (C) ROC curve for working conditions, (D) ROC curve for race, and (E) ROC curve for income.

**Figure 4:**
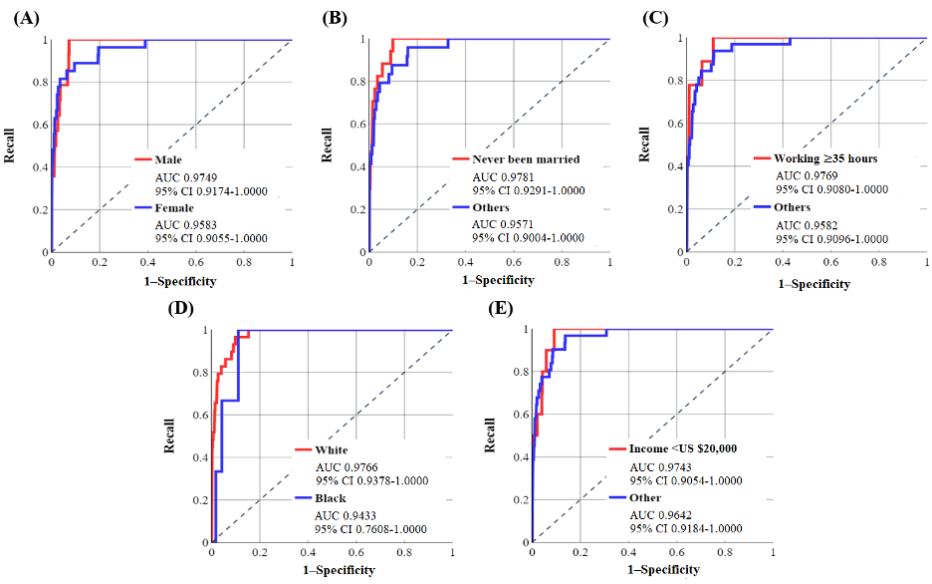
The receiver operating characteristic (ROC) curves for various groups related to sociodemographic features using the neural network model using Adam (with area under the curve [AUC] and 95% CI values). Values were calculated based on the test sample (6579 individuals: 41 developed opioid use disorder [OUD] and 6538 did not develop OUD). (A) ROC curve for sex, (B) ROC curve for marital status, (C) ROC curve for working condition, (D) ROC curve for race, and (E) ROC curve for income.

Figures 3 and 4 demonstrate the ROC curves for these abovementioned sociodemographic groups after training the NN-SGD and NN-Adam, respectively. Tables 3 and 4 show the performance metrics using the default threshold (50%) with *P* values for the difference between the specificity and recall.

According to Table 3, there is a high difference between the AUC values for both groups related to each of the 5 sociodemographic features, and the algorithmic bias was present in the NN-SGD [39]. Furthermore, the *P* values with 95% CI indicate that there is a statistically significant difference between specificity and recall values using various thresholds in the range (0, 100).

According to Table 4, the difference between AUC values for sociodemographic groups is high for all 5 sociodemographic features. Moreover, the accuracy and specificity values are notably high for all groups, and recall values are >57% (except for the Black race, which is 33.33%), which shows the high performance of the model in correctly identifying those with OUD. Similar to the NN-SGD, the *P* values are quite significant, and algorithmic bias is present in the NN-Adam.

The results of detecting algorithmic bias using LR, linear SVM, and SVM-RBF classifiers are presented in Figures S3-S5 in Multimedia Appendix 2, respectively. Tables S1-S3 in Multimedia Appendix 2 also show the performance of these classifiers for sociodemographic features. All the classifiers indicate algorithmic bias. Moreover, while the SVM-RBF classifier indicates the highest bias for race, LR and linear SVM classifiers show a higher bias for sex and marital status.

**Figure 5:**
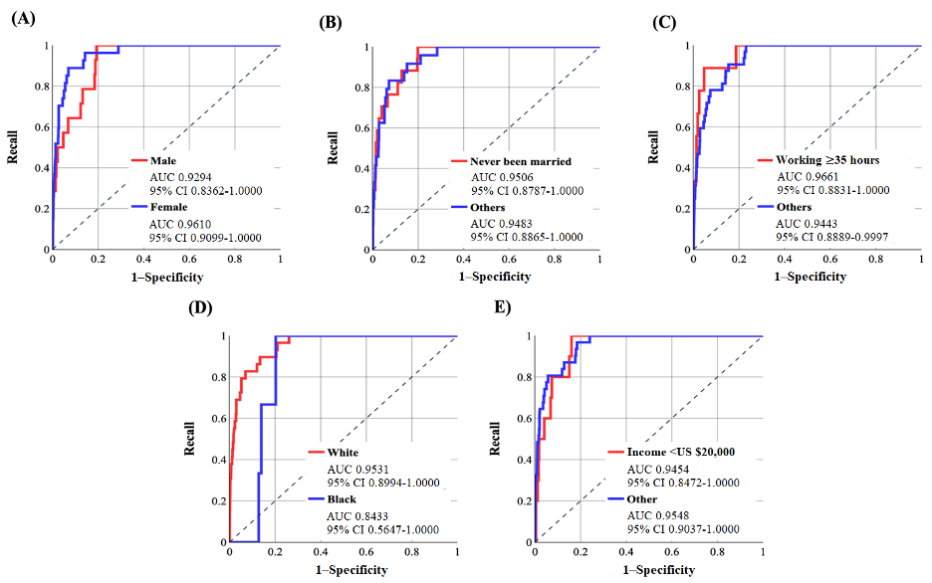
The receiver operating characteristic (ROC) curves for various groups related to sociodemographic features were used using the neural network model using stochastic gradient descent after matching (with area under the curve [AUC] and 95% CI values). Values were calculated based on the test sample (6583 individuals: 41 developed opioid use disorder [OUD] and 6542 did not develop OUD). (A) ROC curve for sex, (B) ROC curve for marital status, (C) ROC curve for working conditions, (D) ROC curve for race, and (E) ROC curve for income.

**Figure 6:**
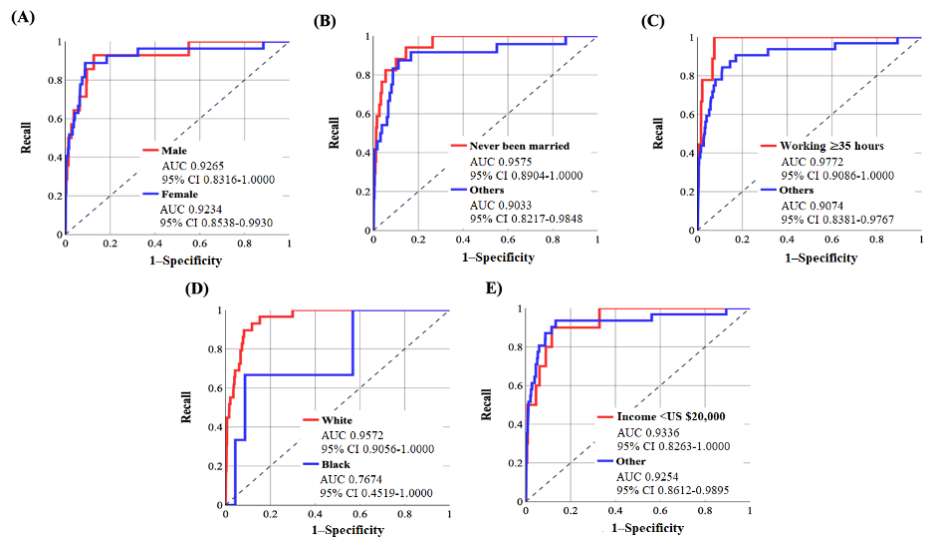
The receiver operating characteristic (ROC) curves for various groups related to sociodemographic features were used using the neural network model using Adam after matching (with area under the curve [AUC] and 95% CI values). Values were calculated based on the test sample (6583 individuals: 41 developed opioid use disorder [OUD] and 6542 did not develop OUD). (A) ROC curve for sex, (B) ROC curve for marital status, (C) ROC curve for working condition, (D) ROC curve for race, and (E) ROC curve for income.

Figures 5 and 6 demonstrate the ROC curves for sociodemographic groups after doing 1-N matching for the NN-SGD and NN-Adam, respectively. Tables 5 and 6 show the performance metrics using the default threshold (50%) with *P* values for the difference between the specificity and recall.

According to Table 5, there is a high difference between the AUC values for each sociodemographic feature, highlighting the existence of algorithmic bias in the NN-SGD [39]. Furthermore, there is a statistically significant difference between specificity and recall values according to *P* values.

According to Table 6, the difference between AUC values for sociodemographic groups is high for all 5 sociodemographic features. Moreover, the accuracy and specificity values are >53% for all groups, except for the Black race, demonstrating the high performance of the model in correctly identifying those with OUD. Furthermore, the *P* values are statistically significant, indicating the existence of algorithmic bias in the NN-Adam.

**Table 3:** The performance metrics of the neural network model using stochastic gradient descent using the default threshold (50%).

Sociodemographic groups		Recall	Specificity	Accuracy	AUC^a^	Difference^b^		*P* value	
**Sex**							22.74		<.001
	Male	7.14	99.60	99.17	82.42				
	Female	29.63	99.35	98.83	87.30				
**Marital status**							3.07		<.001
	Never been married	23.53	99.25	98.80	82.83				
	Other groups of marital status	20.83	99.62	99.12	87.61				
**Working condition**							14.40		<.001
	Working ≥35 hours	11.11	99.77	99.47	92.97				
	Other groups of working condition	25.00	99.26	98.65	81.93				
**Race**							28.08		<.001
	White	27.59	99.55	99.07	99.07				
	Black	0.00	99.06	98.60	98.60				
**Income**							29.93		<.001
	An income of <US $20,000	0.00	98.70	97.72	84.90				
	Other groups of income	29.03	99.60	99.21	85.22				

^a^AUC: area under the curve.

^b^Difference between recall and specificity values.

**Table 4:** The performance metrics of the neural network model using Adam using the default threshold (50%).

Sociodemographic groups			Recall		Specificity		Accuracy		AUC^a^		Difference^b^			*P* value	
**Sex**												21.31			<.001
	Male	57.14		97.69		97.50		97.49							
	Female	77.78		97.02		96.87		95.83							
**Marital status**												10.40			<.001
	Never been married	76.47		96.98		96.85		97.81							
	Other groups of marital status	66.67		97.58		97.39		95.71							
**Working condition**												9.71			<.001
	Working ≥35 hours	77.78		97.73		97.67		97.69							
	Other groups of working condition	68.75		97.05		96.82		95.82							
**Race**												46.57			<.001
	White	79.31		97.07		96.95		97.66							
	Black	33.33		97.66		97.36		94.33							
**Income**												15.38			<.001
	An income of <US $20,000	80.00		94.68		94.54		97.43							
	Other groups of income	67.74		97.80		97.63		96.42							

^a^AUC: area under the curve.

^b^Difference between recall and specificity values.

**Table 5:** The performance metrics of the neural network model using stochastic gradient descent using the default threshold (50%) after matching.

Sociodemographic groups		Recall	Specificity	Accuracy	AUC ^a^	Difference^b^		*P* value	
**Sex**							2.02		<.001
	Male	50.00	97.67	97.47	92.94				
	Female	51.85	97.84	97.46	96.10				
**Marital status**							13.02		<.001
	Never been married	58.82	97.74	97.54	95.06				
	Other groups of marital status	45.83	97.77	97.38	94.83				
**Working condition**							19.91		<.001
	Working ≥35 hours	66.67	97.84	97.66	96.61				
	Other groups of working condition	46.88	97.72	97.40	94.43				
**Race**							60.16		<.001
	White	58.62	97.46	97.21	95.31				
	Black	0.00	99.00	98.51	84.33				
**Income**							3.42		<.001
	An income of <US $20,000	50.00	96.54	96.33	94.54				
	Other groups of income	51.61	98.35	98.02	95.48				

^a^AUC: area under the curve.

^b^Difference between recall and specificity values.

**Table 6:** The performance metrics of the neural network model using Adam using the default threshold (50%) after matching.

Sociodemographic groups		Recall	Specificity	Accuracy	AUC^a^	Difference^b^		*P* value	
**Sex**							2.82		<.001
	Male	57.14	96.80	96.63	92.65				
	Female	59.26	96.10	95.79	92.34				
**Marital status**							11.12		<.001
	Never been married	64.71	96.74	96.58	95.75				
	Other groups of marital status	54.17	96.16	95.84	90.33				
**Working condition**							25.25		<.001
	Working ≥35 hours	77.78	96.00	95.90	97.72				
	Other groups of working condition	53.13	96.60	96.32	90.74				
**Race**							67.63		<.001
	White	65.52	95.90	95.71	95.72				
	Black	0.00	98.01	97.52	76.74				
**Income**							5.40		<.001
	An income of <US $20,000	60.00	94.14	93.99	93.36				
	Other groups of income	58.06	97.60	97.32	92.54				

^a^AUC: area under the curve.

^b^Difference between recall and specificity values.

### Bias Detection (Sampling Bias)

**Figure 7:**
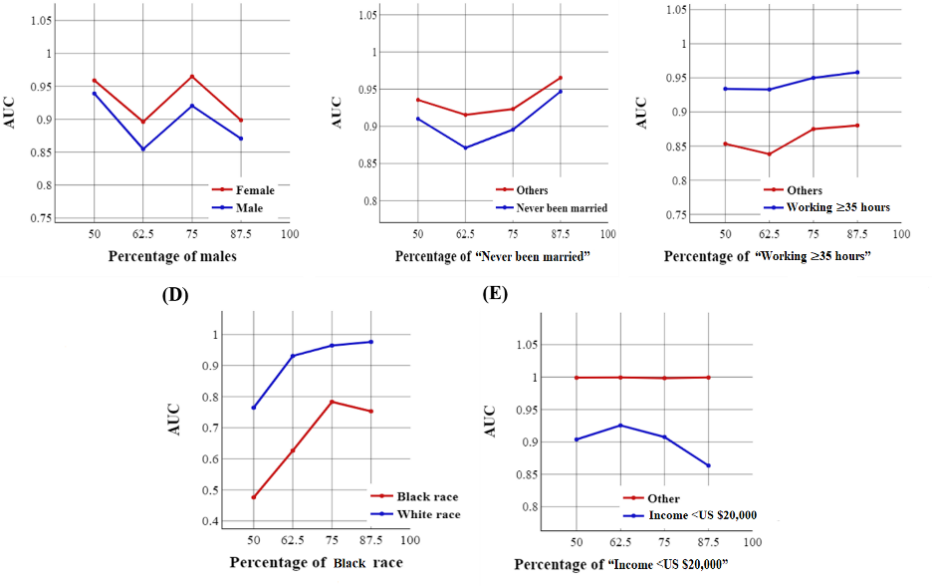
The trend of area under the curve (AUC) values for sociodemographic features using the neural network model using stochastic gradient descent: (A) the trend for sex, (B) the trend for marital status, (C) the trend for working conditions, (D) the trend for race, and (E) the trend for income.

**Figure 8:**
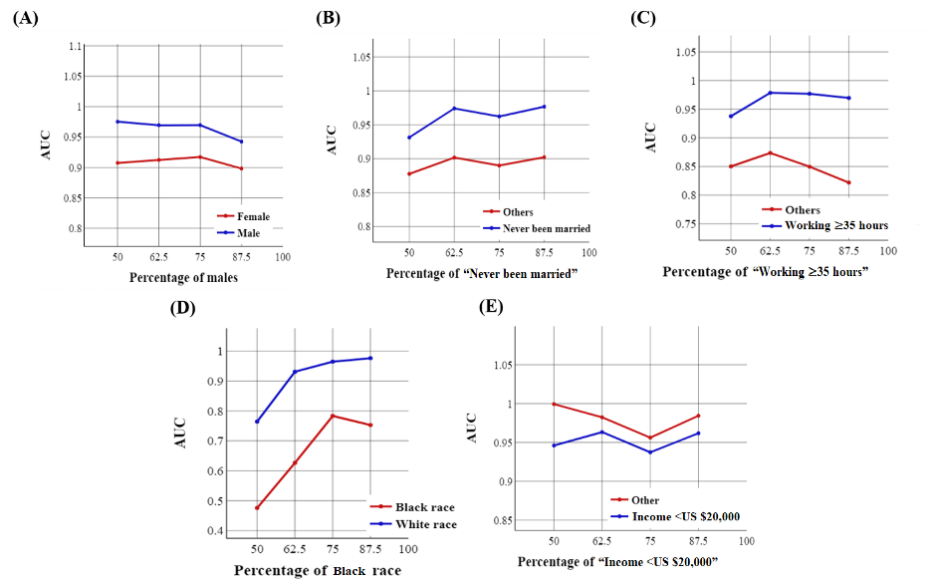
The trend of area under the curve (AUC) values for sociodemographic features using the neural network model using Adam: (A) the trend for sex, (B) the trend for marital status, (C) the trend for working conditions, (D) the trend for race, and (E) the trend for income.

Figures 7 and 8 demonstrate the trend of AUC values based on the structure of the training set using the NN-SGD and NN-Adam, respectively.

We observed significant fluctuations in AUC values for all the demographic groups, especially using the NN-SGD, indicating the presence of sampling bias for all sociodemographic features.

The detection of sampling bias for ML classifiers (ie, LR and SVM) is presented in Figures S6-S8 in Multimedia Appendix 2. The LR and linear SVM classifiers demonstrate a significant sampling bias for all sociodemographic features. In addition, while the SVM-RBF classifier did not show any significant sampling bias for sex, marital status, and working condition, it indicated a notable bias for race and income.

**Figure 9:**
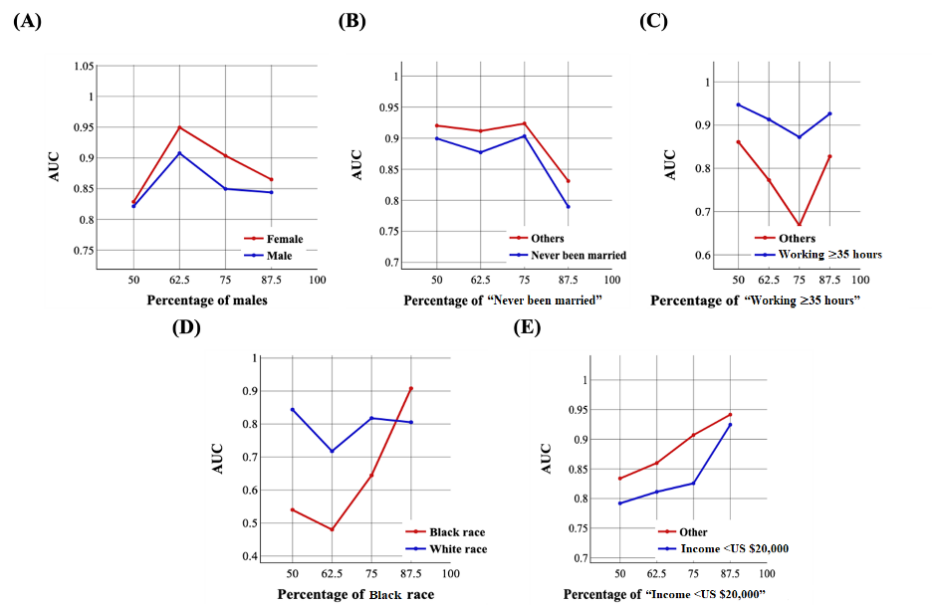
The trend of area under the curve (AUC) values for sociodemographic features using the neural network model using stochastic gradient descent after matching: (A) the trend for sex, (B) the trend for marital status, (C) the trend for working conditions, (D) the trend for race, and (E) the trend for income.

**Figure 10:**
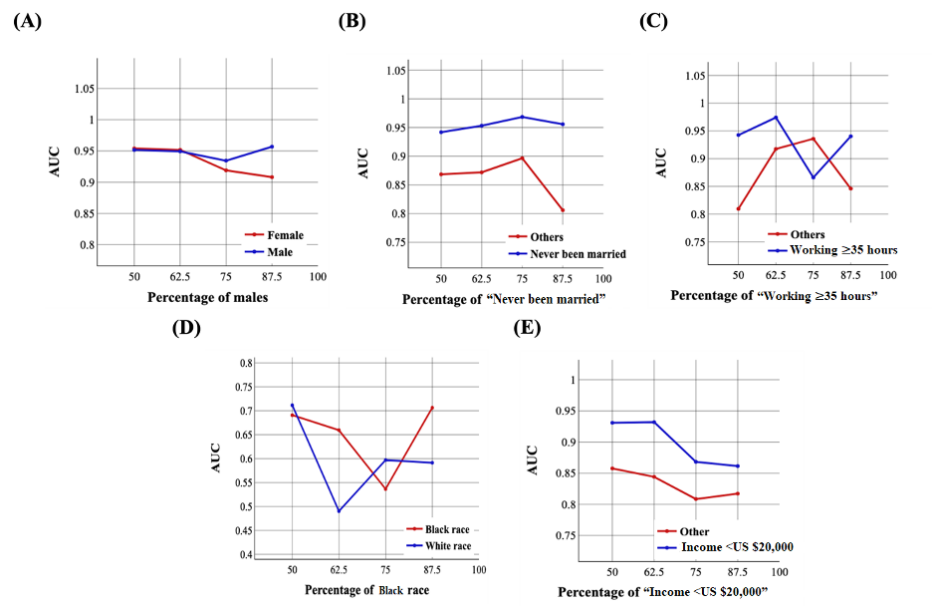
The trend of area under the curve (AUC) values for sociodemographic features using the neural network model using Adam after matching: (A) the trend for sex, (B) the trend for marital status, (C) the trend for working conditions, (D) the trend for race, and (E) the trend for income.

Figures 9 and 10 demonstrate the trend of AUC values based on the structure of the training set using the NN-SGD and NN-Adam after matching, respectively.

We observed significant variations in AUC values, especially using the NN-SGD after matching, highlighting the existence of sampling bias for all sociodemographic features.

### Bias Mitigation

The details of implementing the bias mitigation algorithm, including the optimal threshold and performance metrics, are presented in Tables 7 and 8.

We observed that the recall values for all 5 sociodemographic groups have increased compared to the original NN-SGD using the default threshold of 50%. A similar increase in recall was observed compared to the original NN-Adam, except for *working ≥35 hours* and *income* groups. However, the specificity and accuracy values have decreased for all these groups. Most importantly, the difference between specificity and recall values has decreased for all groups, except the marital status and working condition using the NN-SGD. The reason why the difference has not decreased for the marital status and working condition is that the algorithm enforces the model to have a recall of ≥70% and an accuracy of ≥50%, and thus, it could not find such a threshold after searching all the available options in the range [0,100]. These bias improvements using NN-SGD and NN-Adam for different sociodemographic features are as follows, respectively: sex (21.66% vs 16.96%), marital status (0.00 vs 8.87%), working condition (0.00 vs 8.45%), race (1.48% vs 41.62%), and income (21.04% vs 0.20%).

The improvement in performance of other ML classifiers after implementing our proposed bias mitigation algorithm is presented in Tables S4-S6 in Multimedia Appendix 2. The results indicate that the algorithm is able to mitigate the bias for all sociodemographic features and improve the recall of the model at the same time. It is notable that using LR and linear SVM classifiers, the recall values did not improve for working condition and income, whereas using the SVM-RBF classifier, the recall values improved for all features. The details regarding the improvements gained by this algorithm are presented in Table S7 in Multimedia Appendix 2.

The details of implementing the bias mitigation algorithm after matching, including the optimal threshold and performance metrics, are presented in Tables 9 and 10.

We observed that the recall values for marital status, working condition, and race have increased compared to the original NN-SGD after matching using the default threshold of 50%. A similar increase in recall was observed compared to the original NN-Adam, except for *working >35 hours* as one of the working condition groups. By contrast, sex and income groups achieved higher specificity and accuracy after matching compared to the original NN-SGD and NN-Adam with a 50% threshold. Notably, the difference between specificity and recall values has decreased for all groups. These bias improvements using NN-SGD and NN-Adam after matching for different sociodemographic features are as follows, respectively: sex (0.14% vs 0.97%), marital status (12.95% vs 10.33%), working condition (14.79% vs 15.33%), race (60.13% vs 41.71%), and income (0.35% vs 2.21%).

**Table 7:** The details of implementing bias mitigation for the neural network model using stochastic gradient descent.

Sociodemographic conditions			Optimal threshold		Recall		Specificity		Accuracy		Difference^a^
**Sex**			18.40								*1.08* ^b^
	Male			*85.71* ^*c*^		58.99		59.12			
	Female			*85.19*		58.43		58.63			
**Marital status**			24.10								3.69
	Never been married			*76.47*		87.13		87.07			
	Other groups of marital status			*75.00*		89.35		89.25			
**Working condition**			24.50								15.22
	Working >35 hours			*66.67*		95.01		94.91			
	Other groups of working condition			*71.88*		85.00		84.90			
**Race**			18.40								*26.60*
	White			*89.66*		58.64		58.85			
	Black			*66.67*		62.25		62.27			
**Income**			17.30								*8.89*
	An income of <US $20,000			*90.00*		44.83		45.28			
	Other groups of income			*87.10*		50.82		51.02			

^a^Difference between recall and specificity after bias mitigation.

^b^Italicized values indicate an improvement compared to the initial values (50% threshold).

**Table 8:** The details of implementing bias mitigation for the neural network model using Adam.

Sociodemographic conditions		Optimal threshold	Recall	Specificity	Accuracy	Difference^a^
**Sex**		22.70				*4.35* ^b^
	Male		*78.5*	95.81	95.73	
	Female		*81.48*	94.37	94.27	
**Marital status**		0.60				*1.53*
	Never been married		*100*	65.43	65.64	
	Other groups of marital status		*100*	66.96	67.17	
**Working condition**		35.40				*1.26*
	Working >35 hours		77.78	96.75	96.68	
	Other groups of working condition		*78.13*	95.84	95.70	
**Race**		5.90				*4.95*
	White		*96.55*	90.11	90.15	
	Black		*100*	88.61	88.66	
**Income**		45.60				*15.18*
	An income of <US $20,000		80	94.48	94.34	
	Other groups of income		67.74	97.40	97.24	

^a^Difference between recall and specificity after bias mitigation.

^b^Italicized values indicate an improvement compared to the initial values (50% threshold).

**Table 9:** The details of implementing bias mitigation for the neural network model using stochastic gradient descent after matching.

Sociodemographic conditions		Optimal threshold	Recall	Specificity	Accuracy	Difference^a^
**Sex**		52.10				*1.88* ^b^
	Male		50.00	97.85	*97.65*	
	Female		51.85	*97.88*	*97.49*	
**Marital status**		5.80				*0.07*
	Never been married		*100.00*	50.26	50.51	
	Other groups of marital status		*100.00*	50.33	50.69	
**Working condition**		12.50				*5.12*
	Working ≥35 hours		*88.89*	89.27	89.27	
	Other groups of working condition		*87.50*	85.54	85.56	
**Race**		8.10				*0.03*
	White		*100.00*	73.39	73.56	
	Black		*100.00*	73.42	73.55	
**Income**		52.60				*3.07*
	An income of <US $20,000		50.00	*96.96*	*96.74*	
	Other groups of income		51.61	*98.42*	*98.09*	

^a^Difference between recall and specificity after bias mitigation.

^b^Italicized values indicate an improvement compared with the initial values (50% threshold).

**Table 10:** The details of implementing bias mitigation for the neural network model using Adam after matching.

Sociodemographic conditions		Optimal threshold	Recall	Specificity	Accuracy	Difference^a^
**Sex**		66.60				*1.85* ^b^
	Male		50.00	*97.88*	*97.68*	
	Female		51.85	*97.88*	*97.49*	
**Marital status**		18.60				*0.79*
	Never been married		*88.24*	88.36	88.36	
	Other groups of marital status		*87.50*	88.31	88.30	
**Working condition**		33.80				*9.92*
	Working ≥35 hours		77.78	93.33	93.24	
	Other groups of working condition		*68.75*	94.22	94.06	
**Race**		19.90				*25.92*
	White		*89.66*	88.27	88.28	
	Black		*66.67*	91.20	91.07	
**Income**		68.00				*3.19*
	An income of <US $20,000		50.00	*97.00*	*96.79*	
	Other groups of income		51.61	*98.58*	*98.25*	

^a^Difference between recall and specificity after bias mitigation.

^b^Italicized values indicate an improvement compared with the initial values (50% threshold).

### The Proposed WMV Classifier

**Figure 11:**
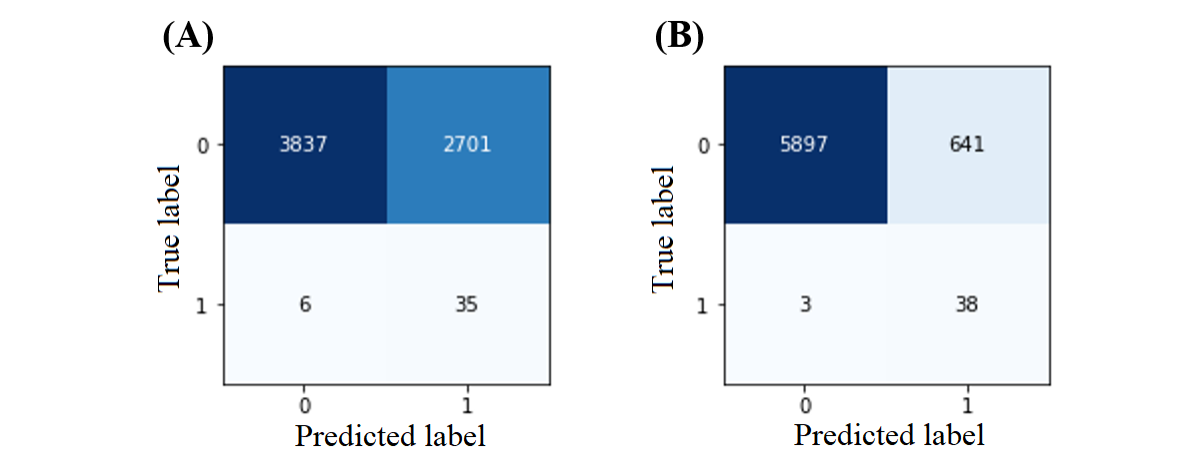
The confusion matrix of the weighted majority voting classifier using neural networks (NNs): (A) NN model using stochastic gradient descent and (B) NN model using Adam.

As mentioned before, we created a WMV classifier and presented its confusion matrices using the NN-SGD and NN-Adam in Figure 11. The feature weights were calculated based on the difference between recall and specificity values using equation 3 based on the default threshold of 50% (Tables 1 and 2). These weights assigned to NN-SGD and NN-Adam for different features are as follows, respectively: sex (0.23 vs 0.21), marital status (0.03 vs 0.10), working condition (0.15 vs 0.09), race (0.29 vs 0.45), and income (0.30 vs 0.15).

The recall of the WMV classifier is >85% using the NNs trained with both optimizers. In addition, while the specificity and accuracy of this classifier using the NN- SGD are approximately 59%, these values are >90% using the NN-Adam. Compared with the NN-SGD and NN-Adam, the WMV classifier has a significantly higher recall; however, the NNs perform better regarding specificity and accuracy because the WMV classifier uses modified thresholds to mitigate the prediction bias. Overall, this WMV classifier that considers the bias issues for all the sociodemographic features has demonstrated satisfactory performance using the NNs trained with SGD and Adam optimizers and can be used for sufficiently accurate and fairness-aware prediction of OUD in individuals.

The weights assigned to each feature and the confusion matrices of the WMV classifier using the ML classifiers are presented in Table S8 and Figure S9 in Multimedia Appendix 2, respectively. According to the results, the recall values of the WMV classifier are higher compared to all the original ML classifiers (>92%). Besides, the specificity and accuracy values are sufficiently high for the WMV classifier using all the ML classifiers (>75%).

**Figure 12:**
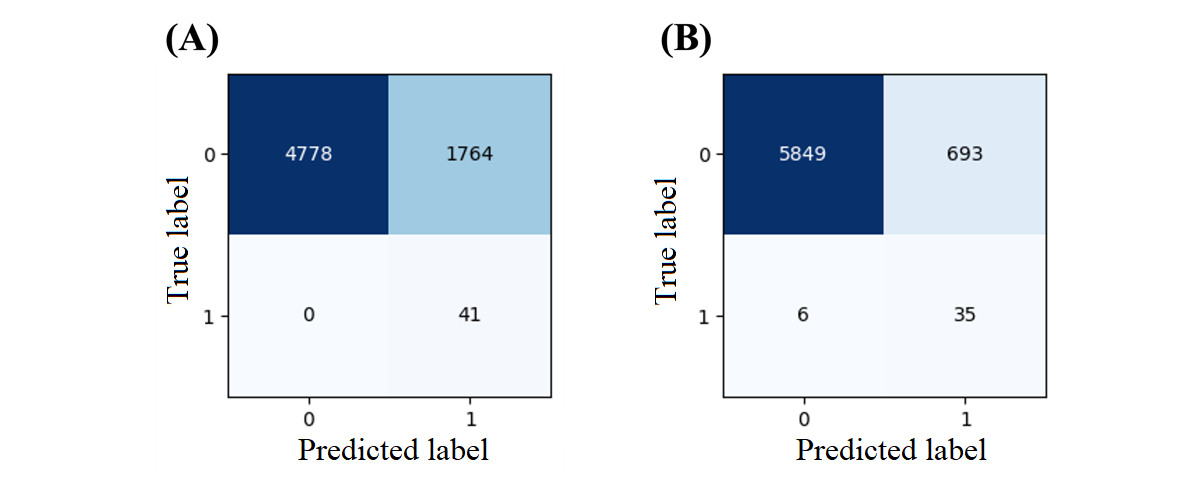
The confusion matrix of the weighted majority voting classifier using neural networks (NNs) after matching: (A) NN model using stochastic gradient descent and (B) NN model using Adam.

Figure 12 shows the confusion matrices of the WMV classifier using the NN-SGD and NN-Adam after matching. The feature weights were calculated based on the difference between recall and specificity values using equation 3 based on the default threshold of 50% (Tables 3 and 4). These weights assigned to NN-SGD and NN-Adam for different features are as follows, respectively: sex (0.02 vs 0.03), marital status (0.13 vs 0.10), working condition (0.20 vs 0.23), race (0.61 vs 0.60), and income (0.03 vs 0.05).

The recall of the WMV classifier is >85% using the NNs trained with both optimizers. In addition, while the specificity and accuracy of this classifier using the NN-SGD are approximately 73%, these values are >89% using the NN-Adam. Compared to the NN-SGD and NN-Adam, the WMV classifier has a significantly higher recall; however, the NNs have higher specificity and accuracy because the WMV classifier uses thresholds for bias mitigation. Overall, this WMV classifier can be used as a fairness-aware predictor of OUD in real-world applications, guiding clinicians in fair and accurate decision-making.

Table 11 demonstrates the performance of different models, including NN-SGD, NN-Adam, and WMV classifiers before and after 1-N matching.

**Table 11:** The performance of different classifiers before and after matching.

Status		Recall	Specificity	Accuracy	Precision
**NN-SGD^a^**					
	Before matching	21.95	99.46	98.98	20.45
	After matching	51.22	97.75	97.46	12.50
**NN-Adam^b^**					
	Before matching	70.73	97.32	97.16	14.22
	After matching	58.54	96.45	96.22	9.38
**WMV^c^ (NN-SGD)**					
	Before matching	85.37	41.31	58.85	1.28
	After matching	100.00	73.04	73.20	2.27
**WMV (NN-Adam)**					
	Before matching	92.68	90.20	90.21	5.60
	After matching	85.37	89.41	89.38	4.81

^a^NN-SGD: neural network model using stochastic gradient descent.

^b^NN-Adam: neural network model using Adam.

^c^WMV: weighted majority voting.

## Discussion

### Principal Findings

According to the results, the proposed bias mitigation algorithm performs well in reducing the bias and producing fairer results for individuals in different sociodemographic groups. However, there is always a trade-off between the bias and the accuracy and specificity of the model. Although the recall values have improved for all sociodemographic groups and the bias has been remarkably mitigated, the accuracy and specificity values have dropped for all these features. Notably, although we could mitigate the bias to a larger extent by solely changing the threshold to minimize the difference, we applied threshold values for accuracy (50%) and recall (70%) so that the overall performance of the model would remain satisfactory. This depends on the user preferences and the importance of bias compared to the model performance in a real-world setting.

The achievements of the proposed bias mitigation algorithm and WMV classifier represent a significant advancement in the field of ML for health care, particularly in addressing fairness and equity in predicting OUD. In the context of health applications, where demographic disparities can lead to unequal treatment outcomes, the ability of this algorithm to substantially reduce bias while enhancing recall is crucial. By trying to equalize recall and specificity across all sociodemographic groups, the algorithm ensures that individuals at risk are equally identified across the groups, which is vital for early and fair intervention and treatment. Compared to existing methods [39,63,64], this approach offers a more rigorous solution considering the performance threshold for both accuracy and recall at the same time. Thus, the classifier maintains an overall satisfactory performance, which is essential in real-world clinical settings where both fairness and accuracy are critical for patient outcomes and sacrificing the performance for achieving fairness is not desirable. These improvements provide a clearer understanding of the impact of the work in real-world health care applications. In addition, unlike many methods that might only mitigate bias for a single feature at a time [39,63,64], our proposed approach mitigates bias for all sociodemographic features, and then, all the results are incorporated into a WMV classifier, making it more viable for deployment in diverse health care environments. Overall, the proposed algorithm and classifier represent a meaningful step forward in creating fairer and more effective ML models for predicting outcomes, such as OUD, thereby potentially improving health equity and treatment efficacy in clinical practice.

The application of the proposed bias mitigation algorithm could be extended far beyond the OUD prediction presented in this study. For example, in the realm of racial bias, the algorithm can be applied to predictive models for cardiovascular disease, ensuring that both Black and White patients receive both equal and accurate risk assessments, thereby improving early detection and treatment for Black patients who might otherwise be overlooked [64]. Similarly, Black women experience a 3 times higher likelihood of mortality from pregnancy-related causes compared to their White counterparts [65], where a biased model could underdiagnose or misdiagnose African Americans, leading to inadequate treatment and poorer health outcomes. In addressing sex bias in heart disease prediction, the algorithm can adjust thresholds to enhance both equality and accuracy for women, ensuring their symptoms are not dismissed and they receive timely care [64]. Moreover, in tackling socioeconomic bias, the algorithm can be used in models predicting the risk of chronic disease, ensuring that individuals from lower-income backgrounds are equally and accurately assessed, leading to equitable health care interventions [66]. In the case of diabetes, where African American populations have higher rates of diabetes compared to non-Hispanic White individuals [67], a biased predictive model might fail to identify at-risk individuals in these minority groups, resulting in delayed diagnosis and treatment. Furthermore, women are more likely to be diagnosed with depression because of societal and sex norms [68], necessitating bias mitigation in predictive models. By addressing these biases while preserving a high performance, the proposed algorithm promotes fairness and improves the reliability of health outcomes predictions across diverse patient populations, making it a valuable tool for enhancing health equity and preventing complications such as cardiovascular issues and eventually death.

After implementing the bias mitigation algorithm, we proposed the WMV classifier to classify the inputs based on the proposed thresholds for sex, marital status, working condition, race, and income. Although this classifier works based on the classification performed for each feature, one may prefer to use the threshold for sex if it is more important than other sociodemographic features in a particular study. This could be the case for other features as well, depending on the user preferences and the type and nature of the study being performed.

In this study, we used SGD and Adam to train the NN models. The reason why we used these 2 optimizers is that we could obtain different bias mitigation results. For example, although the NN-SGD could perform better in mitigating the sex bias compared to NN-Adam (21.66% vs 19.96%), the NN-Adam better mitigated the race bias (41.62% vs 1.48%). Accordingly, one could prefer to use a specific hyperparameter based on the nature of the study and user preferences. For instance, if sex is a more important feature than race in a certain case, Adam optimizer would be a better choice compared to SGD. Overall, we chose the NN-Adam as the final best classifier as it could mitigate the bias for all 5 sociodemographic features and have a higher predictive performance after developing the WMV classifier.

In this study, we observed that the precision is lower than the recall in the WMV classifier. While precision is an important metric that indicates the proportion of true positive predictions among all positive predictions, recall holds higher importance and utility in clinical decision-making [69]. Recall measures the ability of the model to correctly identify all relevant cases, in this instance, true positives among those who actually have OUD. Missing a true positive (ie, a false negative) can have severe consequences, potentially delaying critical treatments or interventions. Therefore, a higher recall ensures that most patients with the condition are identified, even in the presence of high false positives. This trade-off is critical in clinical practice, where the cost of misclassifying a patient at high risk of OUD outweighs the cost of additional testing or follow-up for misclassifying an actual non-OUD patient. Hence, despite the lower precision, the higher recall of our model provides greater overall utility in ensuring patient safety and effective clinical outcomes.

We analyzed the effectiveness of the proposed algorithm using several ML models, including LR, linear SVM, and SVM-RBF. Other ML models exist, such as random forests and decision trees, which could potentially classify OUD with high predictive performance. However, these models do not assign probability values to the output classes, and the proposed algorithm cannot be used to mitigate their potential bias.

### Limitations

This study used the 2020 NSDUH data, with most cases belonging to the non-OUD class and <1% to the OUD class. While it is important to acknowledge this data limitation and contextualize it within the body of research, we used 2 techniques, including class weighting and 1-N matching, to address the class imbalance problem. The experiments following the 1-N matching demonstrated the existence of bias, which was mitigated using the proposed bias mitigation algorithm. Moreover, it is notable that several previous studies have successfully applied ML to predict OUD [5,16,18,19]. Despite being highly imbalanced and including much less positive OUD cases than negative ones, many studies have demonstrated the remarkable potential of ML models for OUD prediction [19]. For example, Hasan et al [18] and Lo-Ciganic et al [16] used credible, real-world claims data to predict OUD with high performance despite their notably high imbalance. Similarly, Han et al [5] used NSDUH data to predict OUD among the US population. These studies demonstrate that, although the data are significantly imbalanced, ML can be effectively used to predict OUD, providing valuable insights and aiding in early intervention strategies.

Although the proposed algorithm works well in removing the bias, some limitations exist in this study. The algorithm can noticeably reduce the bias for a single variable (such as sex) and propose an optimal threshold. However, it cannot suggest a single threshold that best mitigates the bias for a group of variables. Moreover, although we demonstrated the existence of bias for demographic features with multiple groups, our algorithm can consider only 2 different groups at the same time. Furthermore, although we included race as a sociodemographic feature, the number of individuals belonging to the Black race who had developed OUD was very low compared to the White race (3 vs 29), which could degrade the generalization of the classifiers. Therefore, including more individuals from the Black race could improve the reliability of the classifiers in real-world applications.

The proposed bias mitigation algorithm, although effective in reducing bias for OUD prediction, can introduce new forms of bias or overlook specific subpopulations. For instance, within racial categories, specific ethnic subgroups, such as Native Americans or recent immigrants, could be overlooked. These groups might have unique cultural or socioeconomic factors affecting their risk of OUD, leading to biased outcomes if these variations are not captured [70]. Similarly, young adults and older adults might experience OUD differently because of distinct life stages and associated risk factors. Young adults might be more susceptible to peer pressure and experimental substance use, while older adults may have chronic pain issues, leading to prolonged opioid prescriptions [71]. If the model does not adequately capture these age-specific differences, predictions could be less accurate for these groups. In addition, certain groups considered vulnerable such as individuals who have been incarcerated or those experiencing homelessness might not be adequately represented in the survey data. These populations often have higher rates of substance use disorders and face different risk factors compared to the general population. Their exclusion or underrepresentation can result in a model that does not generalize well to these groups, leading to biased predictions. Despite these potential drawbacks, the model offers significant advantages. Systematic adjustment of classification thresholds for existing sociodemographic features ensures balanced predictive performance across different demographic groups, reducing discrimination and improving fairness.

The development and implementation of the WMV classifier enhances the applicability of the proposed bias mitigation algorithm, allowing for tailored threshold adjustments based on the importance of specific sociodemographic features in different studies. This flexibility ensures that the algorithm can be adapted to various contexts, addressing specific fairness concerns as needed. While the WMV classifier performed well in the accurate prediction of OUD, its reliance on proportional importance might not fully capture real-world complexities, such as the interplay between various sociodemographic and health factors. For example, the importance of income might be overestimated, ignoring how low socioeconomic status intersects with other factors such as access to health care and social support networks, thus affecting the model’s accuracy for people from different socioeconomic backgrounds. Educational attainment, geographic location, employment status, occupation types, and housing stability also influence OUD risk and may not be fully accounted for, potentially skewing results and introducing new biases. Continuous evaluation and refinement are necessary to ensure that the model addresses these complexities, minimizing new biases and ensuring equitable outcomes across all populations.

### Conclusions

The OUD is the result of irregular opioid use, which is a significant cause of deaths worldwide. The ML models have great potential in OUD prediction; however, these models are prone to bias because of the existence of sociodemographic features. In this study, we proposed a bias mitigation algorithm based on EO. This algorithm works based on the threshold moving to achieve an optimal threshold, minimizing the difference between the specificity and recall values for sociodemographic groups. In addition, this algorithm considers the threshold for the overall recall and accuracy to ensure that the model performs well in OUD prediction. Finally, we proposed a WMV classifier that makes predictions based on the optimal thresholds for all sociodemographic features. The results suggest that the proposed algorithm achieves 21.66%, 1.48%, and 21.04% bias improvement for sex, race, and income using the NN-SGD. The algorithm using the NN-Adam shows an improvement of 16.96%, 8.87%, 8.45%, 41.62%, and 0.20% for sex, marital status, working condition, race, and income, respectively. This algorithm was also able to increase the recall of these classifiers at the same time. In addition, the WMV classifier achieved recall values of 85.37% and 92.68%, specificity values of 58.69% and 90.20%, and accuracy values of 58.85% and 90.21% using NN-SGD and NN-Adam, respectively. This WMV classifier has the potential to be used as a fairness-aware OUD predictor in a real-world setting. The results of the proposed bias mitigation algorithm and WMV classifier for 3 ML classifiers, including LR, linear SVM, and SVM-RBF, also prove the effectiveness of these methods in bias mitigation and fairness-aware prediction of OUD.

Although this study has achieved its research goals, the recommendations for future research work are as follows. First, the bias mitigation algorithm can be extended by developing a method that considers groups of sociodemographic variables and suggests an optimal global threshold. Second, the algorithm can be extended by developing an approach for mitigating the bias and selecting a threshold value for multigroup sociodemographic features instead of focusing on 2 groups simultaneously. Third, the performance of the bias mitigation algorithm may improve by training the NNs with different hyperparameters, such as the learning rate and optimizer. Fourth, more balanced data containing a higher proportion of samples belonging to the minority class and other sociodemographic features can be used to develop fairness-aware predictive models for real-world applications. Fifth, the proposed methods can be used in other medical applications, including but not limited to disease detection, disease classification, and treatment response prediction.

## Appendix

Multimedia Appendix 1 The details of variables used in the study.

Multimedia Appendix 2 The performance of the proposed bias mitigation algorithm using machine learning classifiers.

## Abbreviations

AUC: area under the curve
DL: deep learning
EO: equality of odds
LR: logistic regression
ML: machine learning
NN: neural network
NN-SGD: neural network model using stochastic gradient descent
NSDUH: National Survey on Drug Use and Health
OD: opioid overdose
OUD: opioid use disorder
PR: precision-recall
RBF: radial basis function
ROC: receiver operating characteristic
SGD: stochastic gradient descent
SVM: support vector machine
WMV: weighted majority voting
