# Supplementary material for: Mitigating Sociodemographic Bias in Opioid Use Disorder Prediction: Fairness-Aware Machine Learning Framework
Source: JMIR AI. 2024 Aug 20;3:e55820. doi: 10.2196/55820 (PMC11372321; doi:10.2196/55820)
Supplement: Multimedia Appendix 1 [file ai_v3i1e55820_app1.docx]

## Appendix 1. The details of variables used in the study.

| **Variable Name^*^** | **Description** | **Type** |
| --- | --- | --- |
| OXCOPDAPYU_1 - Yes | The individual has used Oxycodone. | Binary |
| OXYMPDAPYU_1 - Yes | The individual has used Oxymorphone. | Binary |
| HYDCPDAPYU_1 - Yes | The individual has used Hydrocodone. | Binary |
| HYDMPDAPYU_1 - Yes | The individual has used Hydromorphone. | Binary |
| FENTPDAPYU_1 - Yes | The individual has used Fentanyl. | Binary |
| MORPPDAPYU_1 - Yes | The individual has used Morphine. | Binary |
| CODEPDAPYU_1 - Yes | The individual has used Codeine. | Binary |
| MTDNPDAPYU_1 - Yes | The individual has used Methadone. | Binary |
| TRAMPDAPYU_1 - Yes | The individual has used Tramadol. | Binary |
| BUPRPDAPYU_1 - Yes | The individual has used Buprenorphine. | Binary |
| HEREVER_1 - Yes | The individual has used Heroin. | Binary |
| HEREVER_2 - No | The individual has not used Heroin. | Binary |
| TXEVRRCVD_1 - Yes | The individual has received alcohol or drug treatment. | Binary |
| TXEVRRCVD_2 - No | The individual has not received alcohol or drug treatment. | Binary |
| DIABETEVR_1 - Yes | The individual has the history of diabetes. | Binary |
| DIABETEVR_2 - No | The individual does not have the history of diabetes. | Binary |
| COPDEVER_1 - Yes | The individual has the history of chronic Bronchitis. | Binary |
| COPDEVER_2 - No | The individual does not have the history of chronic Bronchitis. | Binary |
| CIRROSEVR_1 - Yes | The individual has the history of Cirrhosis of the liver. | Binary |
| CIRROSEVR_2 - No | The individual does not have the history of Cirrhosis of the liver. | Binary |
| HEPBCEVER_1 - Yes | The individual has the history of Hepatitis B/C. | Binary |
| HEPBCEVER_2 - No | The individual does not have the history of Hepatitis B/C. | Binary |
| KIDNYDSEV_1 - Yes | The individual has the history of kidney disease. | Binary |
| KIDNYDSEV_2 - No | The individual does not have the history of kidney disease. | Binary |
| ASTHMAEVR_1 - Yes | The individual has the history of Asthma. | Binary |
| ASTHMAEVR_2 - No | The individual does not have the history of Asthma. | Binary |
| HIVAIDSEV_1 - Yes | The individual has the history of AIDS. | Binary |
| HIVAIDSEV_2 - No | The individual does not have the history of AIDS. | Binary |
| CANCEREVR_1 - Yes | The individual has the history of cancer. | Binary |
| CANCEREVR_2 - No | The individual does not have the history of cancer. | Binary |
| ADDPREV_1 - Yes | The patient has the history of depression. | Binary |
| ADDPREV_2 - No | The patient does not have the history of depression. | Binary |
| IRSEX_1 - Male | The individual is male. | Binary |
| IRMARIT_1 - Married | The individual is married. | Binary |
| IRMARIT_2 - Widowed | The individual is widowed. | Binary |
| IRMARIT_3 - Divorced or Separated | The individual is divorced or separated. | Binary |
| IRMARIT_4 - Never Been Married | The individual has never been married. | Binary |
| WRK35WKUS_1 - Yes | The individual works 35 hours or more per week. | Binary |
| WRK35WKUS_2 - No | The individual does not work 35 hours or more per week. | Binary |
| NEWRACE2_White | The individual belongs to the white race. | Binary |
| NEWRACE2_Black | The individual belongs to the black race. | Binary |
| NEWRACE2_Others | The individual belongs to other race groups. | Binary |
| INCOME_1 – Less than $20,000 | The individual has an income of less than $20,000 per year. | Binary |
| BMI | Body Mass Index | Continuous |
| UDPYOPI_1 - Yes | The individual is dependent on or has misused opioids. | Binary |

***** For features with multiple categories, we included both “yes” and “no” groups in the data after one-hot encoding. In such cases, when both “yes” and “no” categories are 0, it means that an individual belongs to any other group. These groups in our data were related to those who did not answer the question or legitimate skip of the answer.
