# Supplementary material for: Mitigating Sociodemographic Bias in Opioid Use Disorder Prediction: Fairness-Aware Machine Learning Framework
Source: JMIR AI. 2024 Aug 20;3:e55820. doi: 10.2196/55820 (PMC11372321; doi:10.2196/55820)
Supplement: Multimedia Appendix 2 [file ai_v3i1e55820_app2.docx]

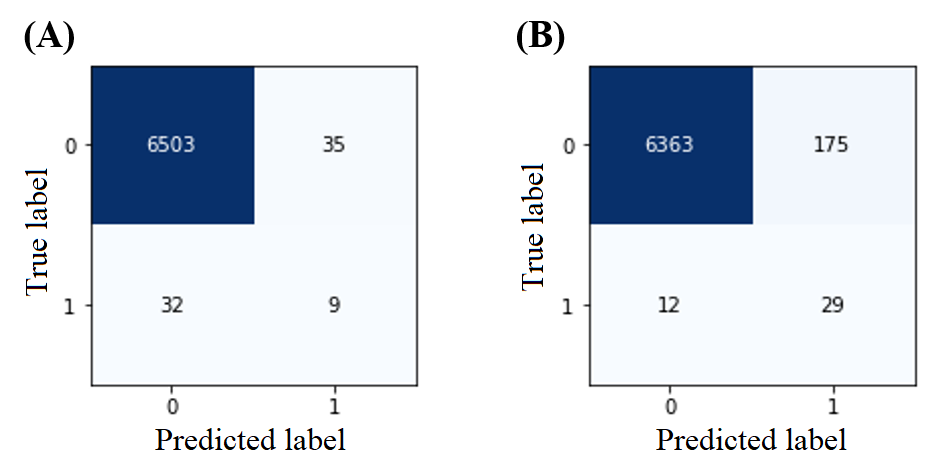


**Figure S1.** The confusion matrix of NNs for OUD prediction. (A) NN-SGD. (B) NN-Adam.

**
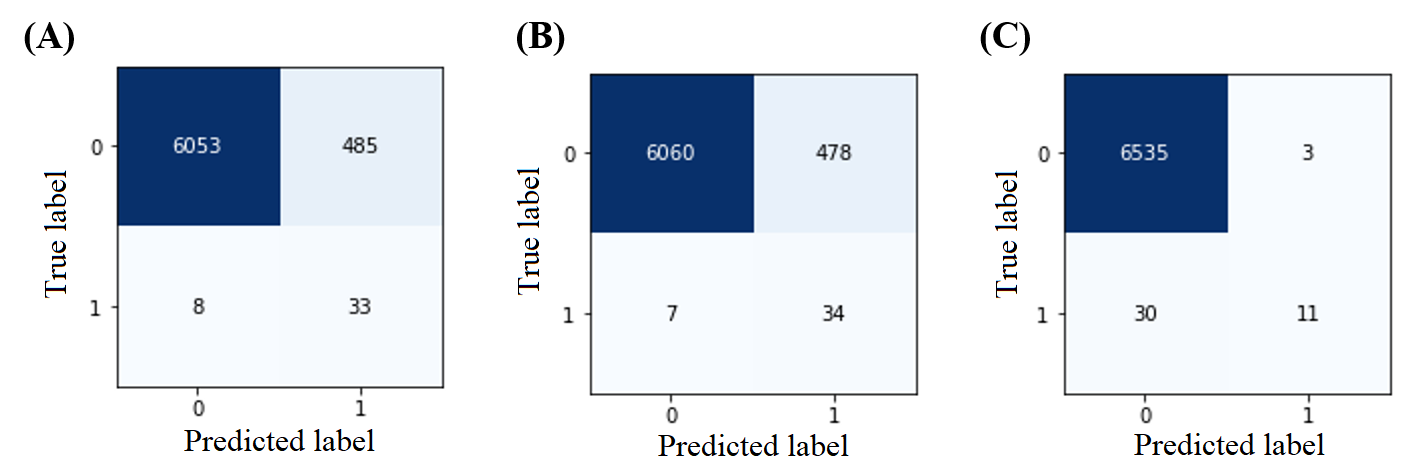
**

**Figure S2.** The confusion matrix of classifiers for OUD prediction. (A) LR classifier. (B) Linear SVM classifier. (C) SVM-RBF classifier.

**
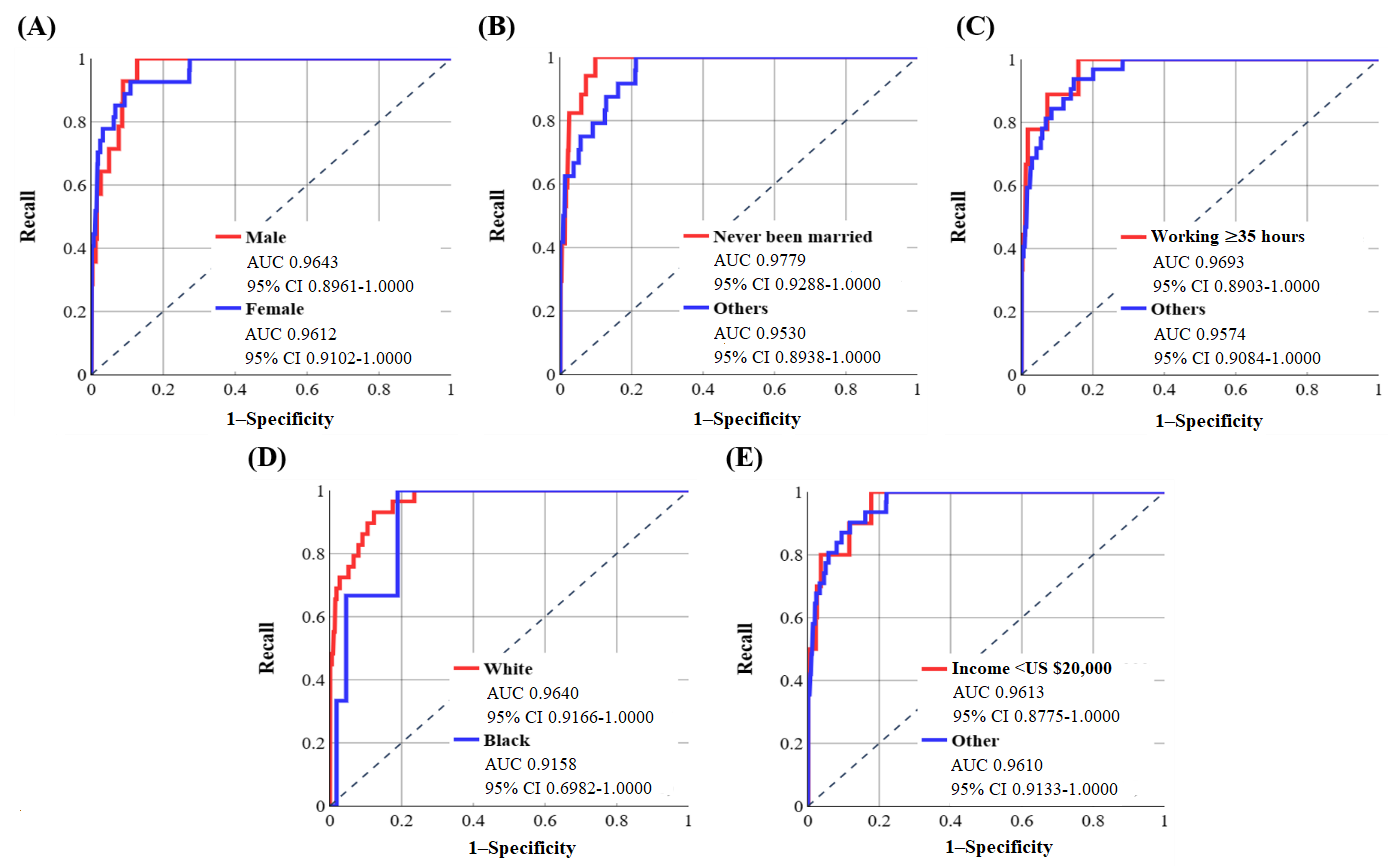
Figure S3.** The ROC curves for various groups related to sociodemographic features using the LR classifier (With AUC values and 95% CIs). Values calculated based on the test sample (6579 individuals: 41 developed OUD, 6538 did not develop OUD). (A) ROC curve for gender. (B) ROC curve for marital status. (C) ROC curve for working condition. (D) ROC curve for race. (E) ROC curve for income.


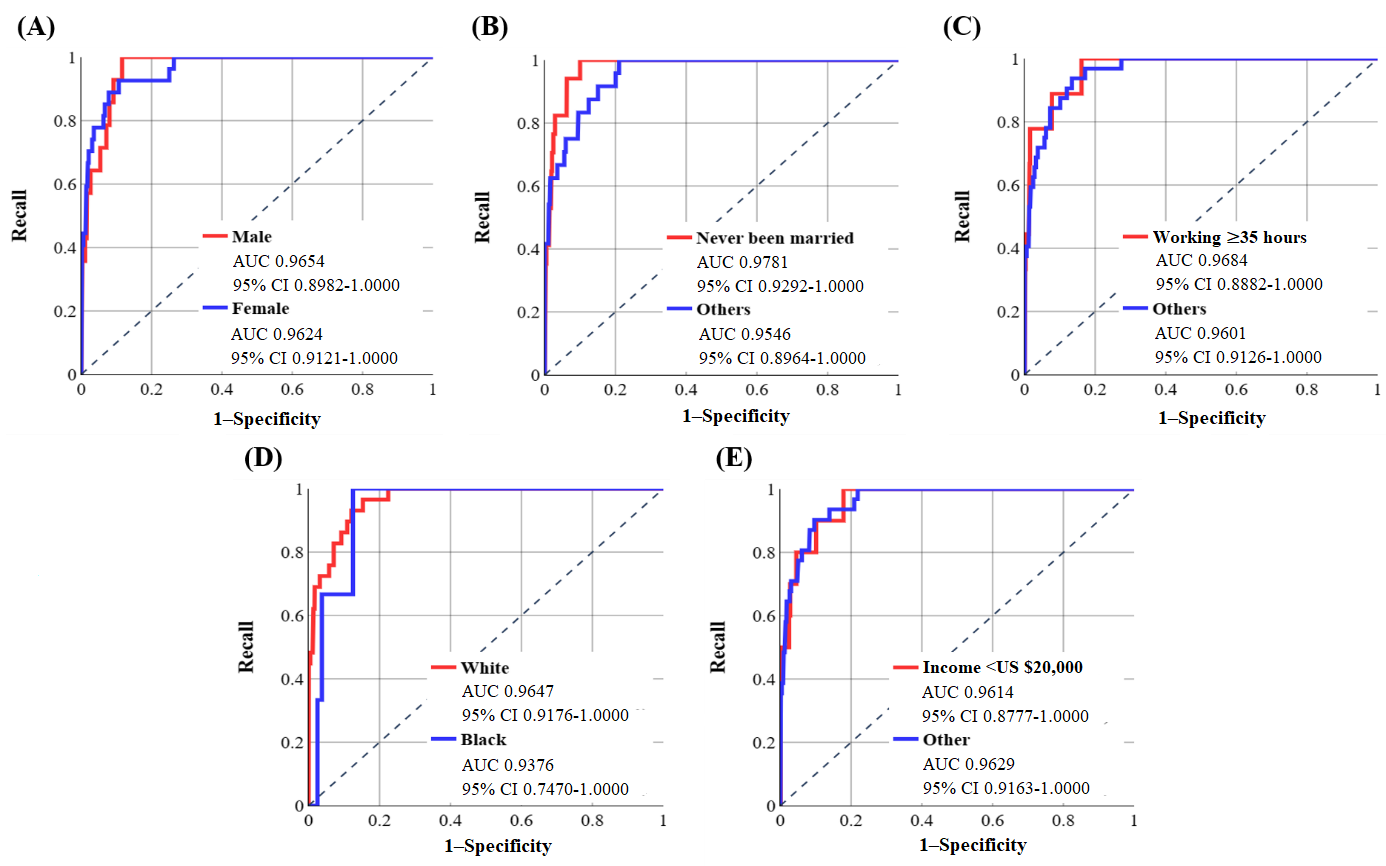


**Figure S4.** The ROC curves for various groups related to sociodemographic features using the linear SVM classifier (With AUC values and 95% CIs). Values calculated based on the test sample (6579 individuals: 41 developed OUD, 6538 did not develop OUD). (A) ROC curve for gender. (B) ROC curve for marital status. (C) ROC curve for working condition. (D) ROC curve for race. (E) ROC curve for income.

**
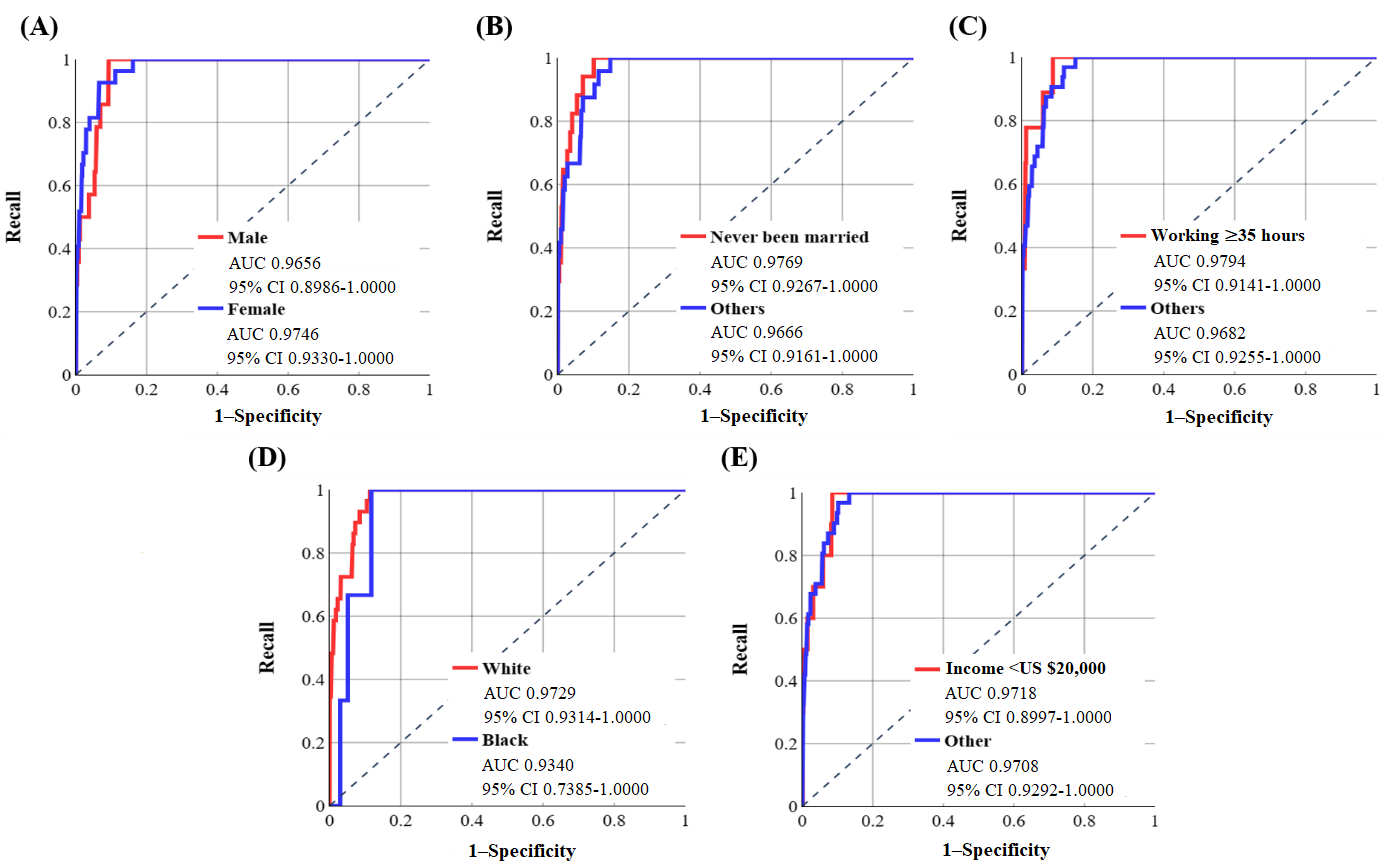
**

**Figure S5.** The ROC curves for various groups related to sociodemographic features using the SVM-RBF classifier (With AUC values and 95% CIs). Values calculated based on the test sample (6579 individuals: 41 developed OUD, 6538 did not develop OUD). (A) ROC curve for gender. (B) ROC curve for marital status. (C) ROC curve for working condition. (D) ROC curve for race. (E) ROC curve for income.


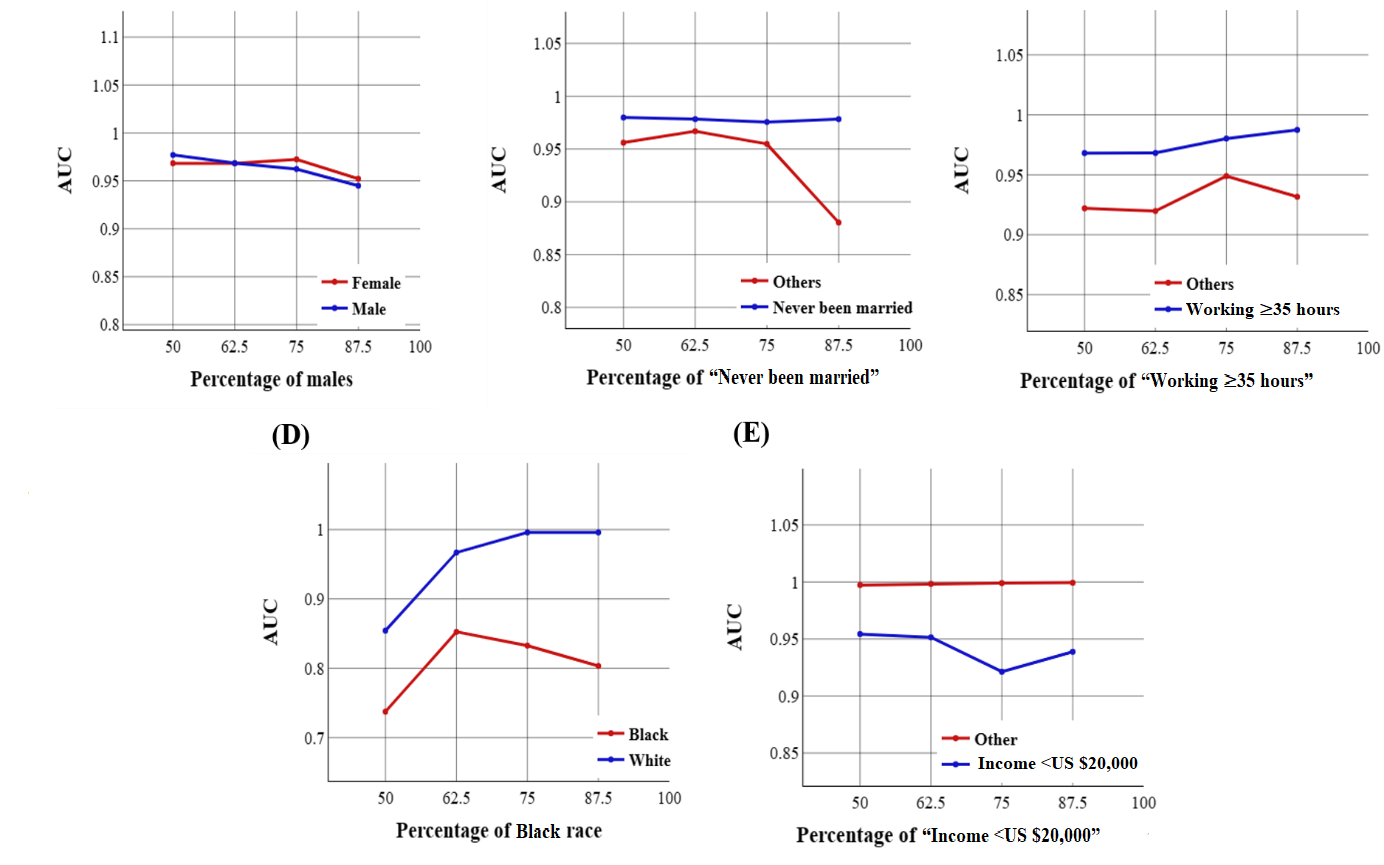


**Figure S6.** The trend of AUC values for sociodemographic features using the LR classifier. Values calculated based on the test sample (6579 individuals: 41 developed OUD, 6538 did not develop OUD). (A) The trend for gender. (B) The trend for marital status. (C) The trend for working condition. (D) The trend for race. (E) The trend for income.

**
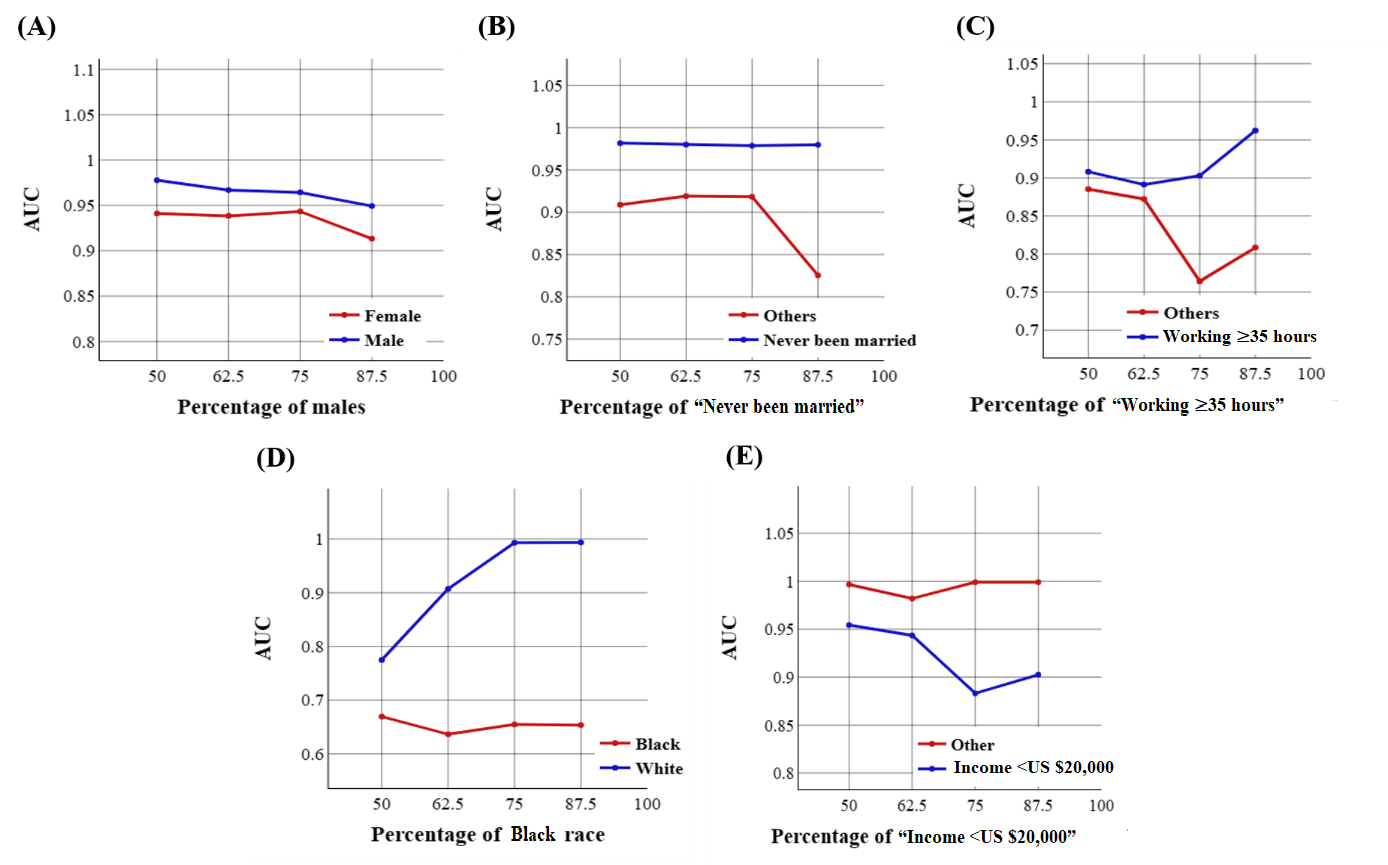
**

**Figure S7.** The trend of AUC values for sociodemographic features using the linear SVM classifier. Values calculated based on the test sample (6579 individuals: 41 developed OUD, 6538 did not develop OUD). (A) The trend for gender. (B) The trend for marital status. (C) The trend for working condition. (D) The trend for race. (E) The trend for income.


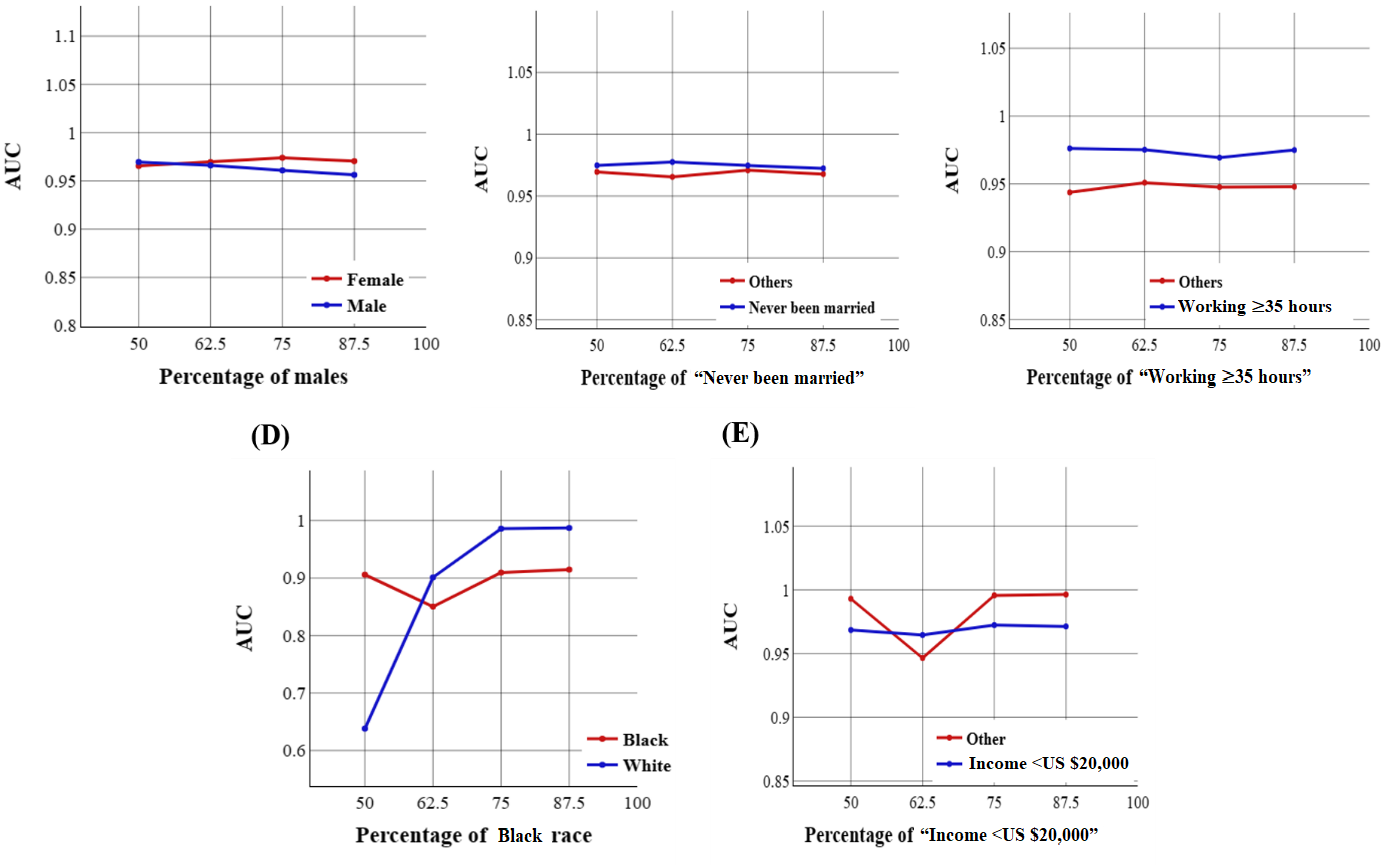


**Figure S8.** The trend of AUC values for sociodemographic features using the SVM-RBF classifier. Values calculated based on the test sample (6579 individuals: 41 developed OUD, 6538 did not develop OUD). (A) The trend for gender. (B) The trend for marital status. (C) The trend for working condition. (D) The trend for race. (E) The trend for income.

**
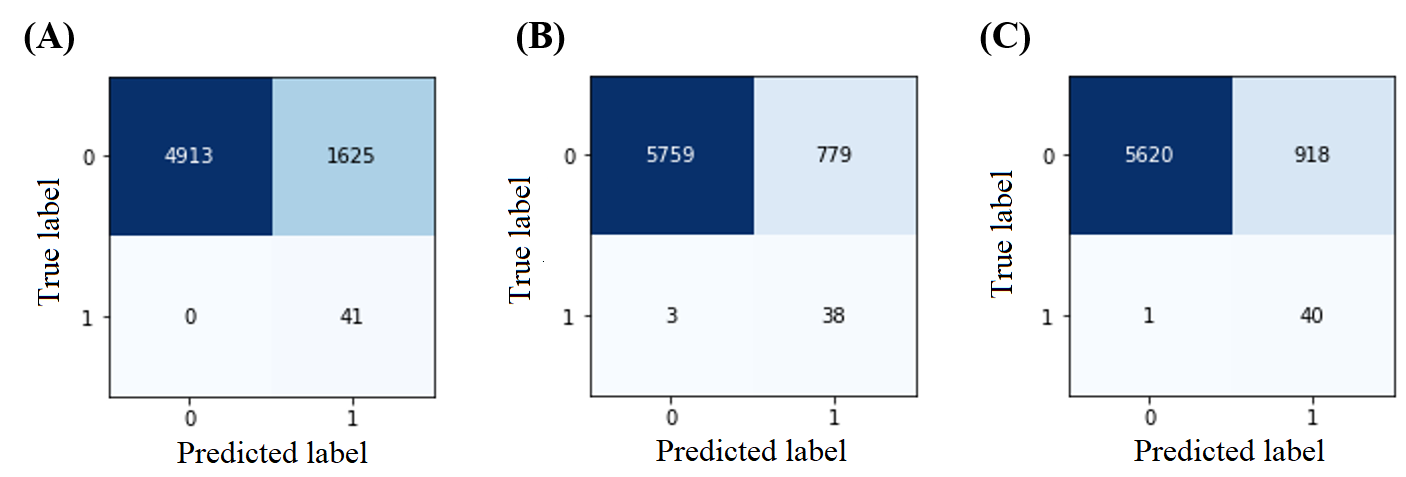
**

**Figure S9.** The confusion matrix of the MV classifier using ML classifiers. (A) LR classifier. (B) Linear SVM classifier. (C) SVM-RBF classifier.

**Table S1.** The performance metrics of the LR classifier using the default threshold (50%).

|  | **Recall** | **Specificity** | **Accuracy** | **AUC** | **Difference** | **P-value** |
| --- | --- | --- | --- | --- | --- | --- |
| **Male** | 71.43 | 94.47 | 94.36 | 96.43 | 17.24 | 0.00 |
| **Female** | 85.19 | 90.99 | 90.95 | 96.12 |  |  |
| **Never Been Married** | 88.24 | 93.07 | 93.04 | 97.79 | 14.09 | 0.00 |
| **Other groups of marital status** | 75.00 | 92.22 | 92.11 | 95.30 |  |  |
| **Working 35 hours or more** | 77.78 | 93.76 | 93.71 | 96.93 | 5.45 | 0.00 |
| **Other groups of working condition** | 81.25 | 91.78 | 91.70 | 95.74 |  |  |
| **White** | 79.31 | 92.24 | 92.15 | 96.40 | 14.55 | <0.001 |
| **Black** | 66.67 | 90.33 | 90.22 | 91.58 |  |  |
| **Income less than $20,000** | 80.00 | 88.77 | 88.68 | 96.13 | 5.15 | 0.00 |
| **Other** | 80.65 | 93.27 | 93.20 | 96.10 |  |  |

**Table S2.** The performance metrics of the linear SVM classifier using the default threshold (50%).

|  | **Recall** | **Specificity** | **Accuracy** | **AUC** | **Difference** | **P-value** |
| --- | --- | --- | --- | --- | --- | --- |
| **Male** | 71.43 | 94.27 | 94.16 | 96.54 | 20.37 | <0.001 |
| **Female** | 88.89 | 91.36 | 91.34 | 96.24 |  |  |
| **Never Been Married** | 94.12 | 93.24 | 93.25 | 97.81 | 20.09 | <0.001 |
| **Other groups of marital status** | 75.00 | 92.27 | 92.16 | 95.46 |  |  |
| **Working 35 hours or more** | 77.78 | 93.46 | 93.40 | 96.84 | 7.89 | <0.001 |
| **Other groups of working condition** | 84.38 | 92.17 | 92.10 | 96.01 |  |  |
| **White** | 82.76 | 92.36 | 92.29 | 96.47 | 18.28 | <0.001 |
| **Black** | 66.67 | 90.17 | 90.06 | 93.76 |  |  |
| **Income less than $20,000** | 90.00 | 88.97 | 88.98 | 96.14 | 13.74 | <0.001 |
| **Other groups of income** | 80.65 | 93.36 | 93.29 | 96.29 |  |  |

**Table S3.** The performance metrics of the SVM-RBF classifier using the default threshold (50%).

|  | **Recall** | **Specificity** | **Accuracy** | **AUC** | **Difference** | **P-value** |
| --- | --- | --- | --- | --- | --- | --- |
| **Male** | 21.43 | 99.93 | 99.57 | 96.56 | 8.24 | <0.001 |
| **Female** | 29.63 | 99.97 | 99.44 | 97.46 |  |  |
| **Never Been Married** | 23.53 | 99.93 | 99.47 | 97.69 | 5.68 | <0.001 |
| **Other groups of marital status** | 29.17 | 99.97 | 99.52 | 96.66 |  |  |
| **Working 35 hours or more** | 22.22 | 99.96 | 99.70 | 97.94 | 5.92 | <0.001 |
| **Other groups of working condition** | 28.13 | 99.95 | 99.36 | 96.82 |  |  |
| **White** | 34.48 | 99.95 | 99.51 | 97.29 | 34.59 | <0.001 |
| **Black** | 0.00 | 99.84 | 99.38 | 93.40 |  |  |
| **Income less than $20,000** | 20.00 | 99.90 | 99.11 | 97.18 | 9.09 | <0.001 |
| **Other groups of income** | 29.03 | 99.96 | 99.57 | 97.08 |  |  |

**Table S4.** The details of implementing bias mitigation for the LR classifier.

|  | **Optimal Threshold** | **Recall** | **Specificity** | **Accuracy** | **Difference** |
| --- | --- | --- | --- | --- | --- |
| **Male** | 27.90 | **92.86** | 91.16 | 91.16 | **7.59** |
| **Female** |  | **92.59** | 83.84 | 83.91 |  |
| **Never Been Married** | 11.80 | **100.00** | 72.33 | 72.50 | **4.94** |
| **Other groups of marital status** |  | **100.00** | 77.27 | 77.41 |  |
| **Working 35 hours or more** | 61.50 | 77.78 | 94.67 | 94.61 | **1.34** |
| **Other groups of working condition** |  | 78.13 | 93.68 | 93.56 |  |
| **White** | 7.80 | **100.00** | 66.75 | 66.98 | **5.44** |
| **Black** |  | **100.00** | 61.31 | 61.49 |  |
| **Income less than $20,000** | 57.70 | 80.00 | 90.37 | 90.27 | **4.52** |
| **Other groups of income** |  | 80.65 | 94.24 | 94.17 |  |

*****The bold numbers indicate an improvement compared to the initial values (50% threshold).

**Table S5.** The details of implementing bias mitigation for the linear SVM classifier.

|  | **Optimal Threshold** | **Recall** | **Specificity** | **Accuracy** | **Difference** |
| --- | --- | --- | --- | --- | --- |
| **Male** | 43.20 | **92.86** | 90.85 | 90.86 | **5.36** |
| **Female** |  | **92.59** | 85.76 | 85.81 |  |
| **Never Been Married** | 35.10 | **100.00** | 73.47 | 73.63 | **5.17** |
| **Other groups of marital status** |  | **100.00** | 78.64 | 78.77 |  |
| **Working 35 hours or more** | 53.70 | 77.78 | 94.70 | 94.65 | **1.24** |
| **Other groups of working condition** |  | 78.13 | 93.81 | 93.68 |  |
| **White** | 36.10 | **100.00** | 77.33 | 77.48 | **5.72** |
| **Black** |  | **100.00** | 71.61 | 71.74 |  |
| **Income less than $20,000** | 51.20 | 80.00 | 89.87 | 89.77 | **4.68** |
| **Other groups of income** |  | 80.65 | 93.90 | 93.83 |  |

*****The bold numbers indicate an improvement compared to the initial values (50% threshold).

**Table S6.** The details of implementing bias mitigation for the SVM-RBF classifier.

|  | **Optimal Threshold** | **Recall** | **Specificity** | **Accuracy** | **Difference** |
| --- | --- | --- | --- | --- | --- |
| **Male** | 0.20 | **100.00** | 73.30 | 73.42 | **3.78** |
| **Female** |  | **100.00** | 69.52 | 69.75 |  |
| **Never Been Married** | 0.30 | **100.00** | 82.04 | 82.15 | **0.01** |
| **Other groups of marital status** |  | **100.00** | 82.05 | 82.16 |  |
| **Working 35 hours or more** | 1.10 | **88.89** | 93.65 | 93.63 | **1.87** |
| **Other groups of working condition** |  | **87.50** | 93.17 | 93.12 |  |
| **White** | 0.40 | **100.00** | 85.04 | 85.14 | **0.17** |
| **Black** |  | **100.00** | 84.87 | 84.94 |  |
| **Income less than $20,000** | 0.30 | **100.00** | 75.23 | 75.47 | **8.04** |
| **Other groups of income** |  | **100.00** | 83.27 | 83.36 |  |

*****The bold numbers indicate an improvement compared to the initial values (50% threshold).

**Table S7.** The improvements in the difference between the recall and specificity values (%).

|  | **Gender** | **Marital Status** | **Working Condition** | **Race** | **Income** |
| --- | --- | --- | --- | --- | --- |
| **LR** | 9.65 | 9.15 | 4.11 | 9.11 | 9.63 |
| **Linear SVM** | 15.01 | 14.92 | 6.65 | 12.56 | 9.06 |
| **SVM-RBF** | 4.46 | 5.67 | 4.05 | 34.42 | 1.05 |

**Table S8.** The weight assigned to each sociodemographic feature for the weighted MV classifier.

|  | **Gender** | **Marital Status** | **Working Condition** | **Race** | **Income** |
| --- | --- | --- | --- | --- | --- |
| **LR** | 0.31 | 0.25 | 0.09 | 0.26 | 0.09 |
| **Linear SVM** | 0.25 | 0.25 | 0.10 | 0.23 | 0.17 |
| **SVM-RBF** | 0.13 | 0.09 | 0.09 | 0.55 | 0.14 |
